# Coordination-driven self-assembly of a molecular figure-eight knot and other topologically complex architectures

**DOI:** 10.1038/s41467-019-10075-6

**Published:** 2019-05-03

**Authors:** Li-Long Dang, Zhen-Bo Sun, Wei-Long Shan, Yue-Jian Lin, Zhen-Hua Li, Guo-Xin Jin

**Affiliations:** Department of Chemistry, Shanghai Key Laboratory of Molecular Catalysis and Innovative Material, State Key Laboratory of Molecular Engineering of Polymers, Fudan University, 2005, Songhu Road, 200438 Shanghai, The People’s Republic of China

**Keywords:** Coordination chemistry, Interlocked molecules

## Abstract

Over the past decades, molecular knots and links have captivated the chemical community due to their promising mimicry properties in molecular machines and biomolecules and are being realized with increasing frequency with small molecules. Herein, we describe how to utilize stacking interactions and hydrogen-bonding patterns to form trefoil knots, figure-eight knots and [2]catenanes. A transformation can occur between the unique trefoil knot and its isomeric boat-shaped tetranuclear macrocycle by the complementary concentration effect. Remarkably, the realization and authentication of the molecular figure-eight knot with four crossings fills the blank about 4_1_ knot in knot tables. The [2]catenane topology is obtained because the selective naphthalenediimide (NDI)-based ligand, which can engender favorable aromatic donor-acceptor π interactions due to its planar, electron-deficient aromatic surface. The stacking interactions and hydrogen-bond interactions play important roles in these self-assembly processes. The advantages provide an avenue for the generation of structurally and topologically complex supramolecular architectures.

## Abstract (web summary)

Molecular knots and links continue to fascinate synthetic chemists. Here, the authors use stacking and hydrogen-bonding interactions between a set of similar building blocks to construct several complex molecular topologies, including a figure-eight knot and a trefoil knot.

## Introduction

Scientists’ interest in complicated molecular knots, links, and entanglements has grown rapidly in recent decades^1–5^. Naturally occurring DNA knots were first discovered in 1967^6,7^, and nearly a decade later, circular DNA-containing links were reported^8^. Carbonic anhydrase was the first identified protein with a knotted tertiary structure, a finding published in 1977^9^. Recently, AFM imaging was employed to unequivocally illustrate the catenane and trefoil knot structures of polymer molecules^10^. While knots and links have particular relevance in the field of biology, they also have been the subject of significant advances in chemical topology. The first nontrivial molecular knot, a trefoil knot^11^, was synthesized by Sauvage using a metal template strategy, along with a Solomon link^12^. Examples of figure-eight knots^13^, pentafoil knots^14–17^, 8_19_ knots^18^, and 8_18_ knots^19^ have since been successfully synthesized. Recently, the realization of a + 3_1_ # + 3_1_ # + 3_1_ composite knot^20^ and a granny knot^21^ have pushed new boundaries in the synthesis of complicated knots. Although a number of trefoil knots (3_1_)^22–28^ have been prepared to date, there are few works providing an insight into the transformation between monomeric macrocycles, molecular knots and links. In addition, in contrast with trefoil knots, figure-eight knots (4_1_) are exceedingly rare. Only one likely synthesis of a molecular figure-eight knot (4_1_) exists, provided by the group of Sanders, the structure of which was determined based largely on symmetry and NMR data^13^. And that the representation of 4_1_ knot is eight crossings rather than four crossings by additional four nugatory crossings^3^. To date no single-crystal structure exists of either a synthetic molecular figure-eight knot or the reduced representation with four crossings, thus our understanding of molecular figure-eight knots remains rudimentary. Thus, constructing and authenticating such a species remains a formidable challenge in the field of supramolecular chemistry. In recent years, the organometallic half-sandwich fragments [Cp*M] (M = Ir, Rh; Cp* = *η*^5^-pentamethyl-cyclopentadienyl) and [Ru(*p*-cymene)] have emerged as versatile building blocks for the construction of supramolecular compounds such as molecular Borromean rings^29^, molecular Solomon links^30^, Hopf’s links^31^, and so on.

**Fig. 1:**
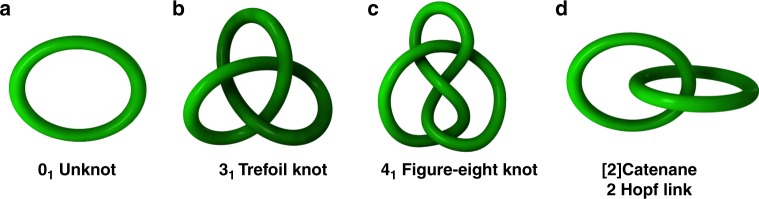
The macrocycles, molecular knots and links prepared in this study with their trivial names and descriptors using the Alexander–Briggs notation^38^. **a** 0_1_ Unknot, (**b**) 3_1_ Trefoil knot (**c**) 4_1_ Figure-eight knot and (**d**) [2]Catenane

Herein, we report the coordination-driven self-assembly^31–37^ of monomeric macrocycles, trefoil knots, figure-eight knots, and [2]catenanes by the combination of flexible ester (**L1**) and amide (**L2**) ligands with [Cp*M] (M = Ir, Rh) organometallic connecting units. Interestingly, the transformation between monomeric macrocycles, trefoil knots and links is effected by merely changing the size of the side arms units. The realization and authentication of a molecular figure-eight knot presented herein is an inspiring and long-awaited achievement. A careful study of single-crystal structure of the knot indicates that the molecule can adopt various forms by altering its conformation, including the reduced form with four crossings and the four-fold symmetry form with eight crossings (Fig. 1). These synthesized knots and links are unambiguously characterized by NMR spectroscopy, ESI-MS, and single-crystal X-ray diffraction analysis. Furthermore, density functional theory (DFT) calculations are used to provide insight into the formation of the [2]catenane and trefoil knots.

## Results

### Selection of ligands

The flexible ligands 1,4-phenylenebis(methylene) diisonicotinate (**L1**) and N,N′-[1,4-phenylenebis-(methylene)]bis-4-pyridinecarboxamide (**L2**) were chosen because of the high degrees of rotational freedom of its ester and amide functional groups, which can allow the ligand to present a variety of configurations and induce hydrogen-bond interactions. In addition, its π-conjugated phenyl and pyridyl moieties can engender favorable aromatic π–π stacking and CH–π interactions^39–41^. The stacking interactions and hydrogen bonding interactions can be considered as the driving force for the formation of trefoil knot and figure-eight knot, while a [2] catenane was formed by the combination of another planar, electron-deficient aromatic edge unit (**E4**) with **L1**.

### Self-assembly of a tetranuclear macrocycle and trefoil knot

**Fig. 2:**
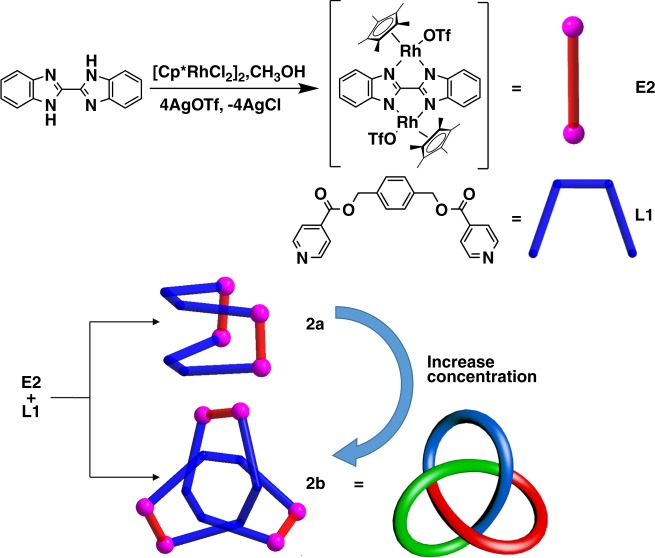
Synthesis of tetranuclear macrocycle **2a** and trefoil knot **2b**. Schematic representation of the synthesis of **E2** and the stick model of **E2**; Schematic representation and stick model of **L1**; Increasing the concentration of **2a** can result in gradual transformation of **2a** into **2b**

**Fig. 3:**
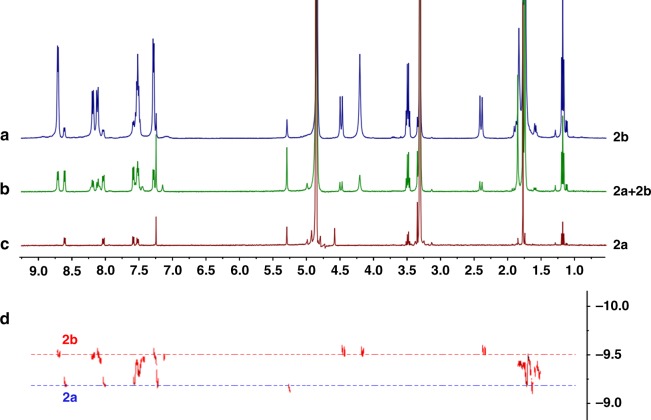
^1^H NMR spectrum (400 MHz, CD_3_OD) of **2b** (**a**), **2a** + **2b** (**b**), and **2a** (**c**). DOSY spectrum (500 MHz, CD_3_OD) of **2a** + **2b** (**d**), (The peaks at 3.50 and 1.18 ppm belong to diethyl ether)

The reaction of [Cp*RhCl_2_]_2_ with AgOTf (2.0 equiv), followed by the addition of **L1**, produced chair-shaped tetranuclear macrocycle complex **1** (yield: 92%) (Supplementary Fig. 12). The structure of **1** was confirmed by electrospray ionization mass spectrometry (ESI-MS), ^1^H NMR spectroscopy, and X-ray crystallographic analysis (Supplementary Fig. 1). The ESI-MS data of **1** in CH_3_OH shows a peak at 2235.04 *m*/*z* assigned to [**1**–OTf^–^]^+^, indicating that complex **1** is stable in solution (Supplementary Fig. 50). Upon treating flexible ligand **L1** with the longer edge unit **E2** in a 1:1 molar ratio, a yellow mixture was obtained in a total yield of 90% (Fig. 2), which was studied by NMR spectroscopy in CD_3_OD (Fig. 3).

Diffusion-ordered NMR spectroscopy (DOSY) indicated the presence of two diffusion coefficients (*D* = 4.7 × 10^−10^ m^2^s^−1^(**2a**) and 2.8 × 10^−10^ m^2^s^−1^ (**2b**)), suggesting the existence of two different compounds in the reaction mixture (Fig. 3). Increasing the concentration of **2a** + **2b** in CD_3_OD from 2.0 mM to 32.0 mM (with respect to Cp*Rh; Supplementary Fig. 28) led to gradual transformation of the tetranuclear complex **2a** into trefoil knot **2b**. At a low concentration (2.0 mM), only **2a** was observed in solution (Supplementary Fig. 18). When in a saturated solution (32.0 mM), **2a** was almost entirely converted to **2b** (90.6 mass%) based on ^1^H NMR spectroscopy (Supplementary Fig. 21). The ^1^H NMR signals of the phenyl and benzyl protons of **2b** showed large upfield shifts to 2.0–4.5 ppm, indicating the tight π–π stacking of phenyl groups. To further study the contribution of π–π stacking interactions to the formation of **2b**, the π-electron-rich guest molecule pyrene was added into the mixture of **2a** + **2b**. Upon addition of increasing amounts of pyrene (from 0 to 6 equiv), the mixture was converted to the pure monomeric macrocycle **2a** based on ^1^H NMR spectroscopy (Supplementary Fig. 32). Based on the established hydrophobic properties of these macrocycles^42–44^, D_2_O was added gradually to the 12.0 mM CD_3_OD solution of **2a** + **2b**. The resulting ^1^H NMR spectrum showed that upon changing the solvent ratio (CD_3_OD:D_2_O, v/v) from 7:0 to 7:7, the mixture of **2a** + **2b** underwent nearly complete transformation to the trefoil knot **2b** (Supplementary Fig. 33). In addition, a ^1^H NMR spectrum in DMSO showed that the vast majority of the complex existed in the monomeric macrocycle **2a** form over a wide concentration range (8.0–24.0 mM, with respect to Cp*Rh; Supplementary Fig. 34).

Along with this NMR spectroscopic data, ESI-MS also indicated the presence of two complexes in solution: [**2a** –OTf^–^]^+^ (*m/z* = 2559.27) (Supplementary Fig. 51) and [**2b**–2OTf^–^]^2+^ (*m/z* = 1882.24) (Supplementary Fig. 52). Single crystals suitable for X-ray diffraction were obtained by slow vapor diffusion of diethyl ether into a methanol solution of **2a** and **2b**, in order to unambiguously confirm the structure and topology of **2a** and **2b**.

**Fig. 4:**
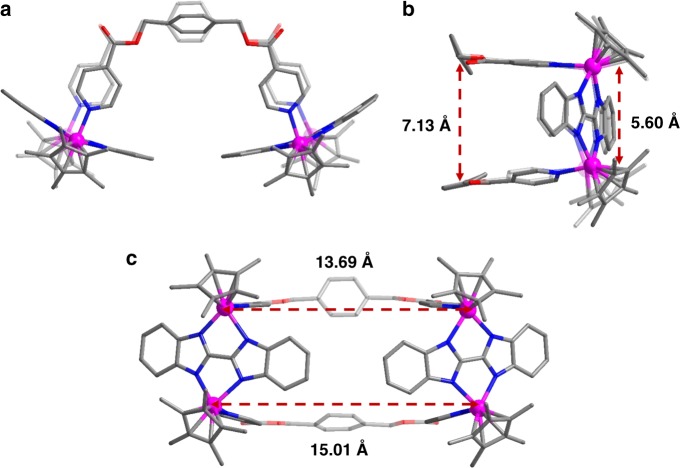
Single-crystal X-ray structure of** 2a**. Top view (**a**), side view (**b**), and front view (**c**). Counteranions and hydrogen atoms are omitted for clarity (N, blue; O, red; C, gray; Rh, purple)

The solid-state structure of complex **2a** was confirmed by single-crystal X-ray diffraction analysis to be a boat-shaped tetranuclear macrocycle. Interestingly, the structure is unsymmetrical, with dimensions of 5.60 Å (short Rh**∙∙∙**Rh nonbonding distance; Fig. 4), 13.69 and 15.01 Å (long Rh**∙∙∙**Rh nonbonding distances; Fig. 4). The distance between the two phenyl groups of **L1** is 7.13 Å, which is even longer than the short Rh**∙∙∙**Rh nonbonding distance of **E2**, indicating that there is no π–π stacking interactions between the phenyl groups.

The crystal structure of **2b** was refined in the *C12/c1* space group, revealing the complex to have the topology 3_1_ according to the Alexander–Briggs notation^38^. The right-handed trefoil knot + 3_1_ has three positive crossings^3,11,24^, and a highly symmetrical main framework of point group *C*_3_. The mirror-image symmetric isomeric topology –3_1_ exists in the same cell in a 1:1 molar ratio. Left-handed trefoil knot –3_1_ has three negative crossings, and a highly symmetrical main framework with the same point group *C*_3_ (Supplementary Fig. 4).

**Fig. 5:**
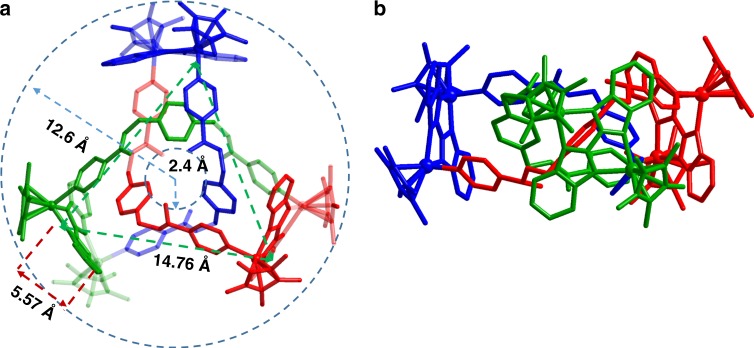
Single-crystal X-ray structure of **2b**. Top view (**a**) and side view (**b**). Counteranions and hydrogen atoms are omitted for clarity

The ligand arms **E2** and **L1**, which are connected by Rh atoms, form a main framework consisting of two triangles, with dimensions of 14.76 and 5.57 Å (Rh**∙∙∙**Rh nonbonding distances; Fig. 5). The average outer diameter of the structure is 24.4 Å and the average inner diameter is 4.8 Å (a circle bound by the inner three O atoms; Fig. 5). A close-contact analysis of the structure shows that the trefoil knot is stabilized by parallel-displaced π–π interactions (of interlayer distance 3.38 Å) between the pyridyl moieties and phenyl moieties of three ligands **L1**, as well as edge-to-face-type CH–π interactions (2.66 Å) between BiBzIm moieties and phenyl moieties (Supplementary Fig. 2). Moreover, in the solid state, intermolecular hydrogen bonds exist between the O atoms of the ester units and the Cp* protons of another contiguous molecular knot, in the range of 2.51 to 2.67 Å (Supplementary Fig. 3). In order to gain insight into the formation of **2b**, DFT binding energy calculations were performed to study the intermolecular interactions in **2b** (trefoil knot; Supplementary Table 1). The energy of formation of the trefoil knot structure from three monomers of **2_3mo** (monomer like **4**; Supplementary Fig. 56) was calculated to be −76.0 kcal/mol, while the contribution energy of π–π stacking was found to be −53.9 kcal/mol.

### Self-assembly of a figure-eight knot (3)

The generation of the trefoil knot **2b** gave us great inspiration, in that stacking interactions and intermolecular hydrogen bonds interactions clearly play synergistic roles in the formation of organometallic knots. Given this realization, in the place of ester-containing ligand **L1**, we decided to employ the amide-containing ligand **L2**. We speculated that the amide groups of **L2** would provide additional means of hydrogen bonding due to the presence of the N–H group, enabling the construction of amide-amide H-bonds and perhaps leading to more extensive hydrogen bonding and more complicated structures.

**Fig. 6:**
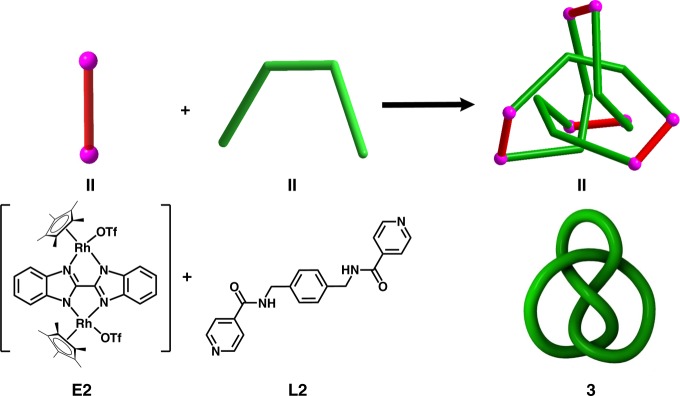
Synthesis of octanuclear figure-eight knot **3**. Schematic representation of the synthesis of **3;** The stick model (top) and schematic representation (below) of **E2** and **L2**; The stick model (top) and simplified image of **3**

A yellow solid **3** (yield: 82%) was obtained by treating amide ligand **L2** with edge unit **E2** in a 1:1 molar ratio (Fig. 6), and the structure of **3** was confirmed by NMR spectroscopy, ESI-MS, and single-crystal X-ray diffraction analysis.

The ^1^H NMR spectrum of **3** in CD_3_OD exhibits two sharp Cp* singlets at *δ* = 1.771 and 1.683 ppm in a ratio of 1:1, corresponding to two disparate Cp*Rh environments, which may signify the existence of a specific topological structure. Furthermore, some signals in the ^1^H NMR spectrum of **3** are shielded with respect to those of building block **E2**, reflecting the compact structure of the molecule, in which each region of the loop is in close proximity with aromatic rings (Supplementary Fig. 35).

The ^1^H DOSY NMR spectrum of **3** (Supplementary Fig. 38) showed that the aromatic and Cp* signals were associated with a single diffusion constant, suggesting that only one stoichiometry of assembly was formed. The structure of **3** in solution was also supported by ESI-MS. The prominent peaks at *m*/*z* = 2555.44 ([3−2OTf^–^]^2+^) is in good agreement with their theoretical distribution (Supplementary Fig. 53), suggesting that the structure remains intact in solution. The ^1^H NMR signals did not change over a wide concentration variation range (2.0–12.0 mM, with respect to Cp*Rh, Supplementary Fig. 39), indicating a compact and stable structure.

**Fig. 7:**
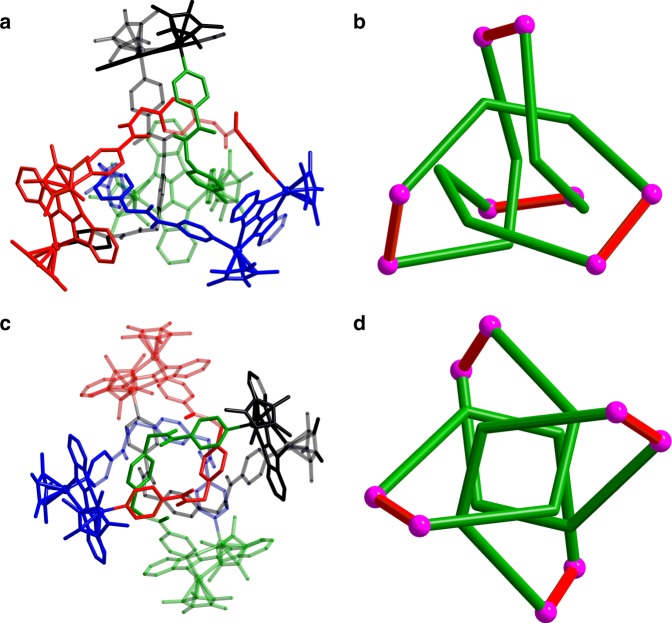
Single-crystal X-ray structure of **3**. The reduced representation with four crossings (**a**) and the four-fold symmetry representation (**c**) of **3**, and simplified structures of the reduced representation with four crossings (**b**) and the four-fold symmetry representation (**d**) of **3** in which sticks connect the rhodium centers

Single crystals of **3** were obtained by slow diffusion of diethyl ether vapor into a solution of **3** in methanol and the solid-state structure was determined by X-ray diffraction analysis. The crystal structure of **3** was refined in the *I4*_*1*_*/a* space group. The crystal structure unequivocally confirmed the topology of the molecular 4_1_ knot according to the Alexander–Briggs notation^38^. (Fig. 7) show the reduced form of the 4_1_ knot comprising four crossings. When viewing the structure **3** in the c direction, the molecular knot **3** is highly symmetrical and has a rotary inversion axis (*S*_4_), which means that a rotation of 90° converts this representation into its mirror image, thereby making it achiral (Fig. 7).

As in trefoil knot structure **2b**, **E2**, and **L2**, which are connected by Rh atoms, form a closed loop with four nonalternating crossings. Close-contact analysis of the structure reveals a figure-eight knot arrangement similar to a tetrahedral configuration, held together by both edge-to-face-type CH–π interactions (3.39 Å) and parallel-displaced π–π interactions (of interlayer distance 3.72 Å) between phenyl moieties and relatively adjacent two pyridyl moieties of three **L2** ligands. Unlike trefoil knot **2b**, in which C–O^**…**^H intermolecular hydrogen bond interactions are formed between carbonyl oxygen atoms of the ester moieties and Cp* protons, the figure-eight knot **3** presents intramolecular N–H^**…**^O hydrogen bonding interactions (2.79 Å) between NH hydrogen atoms and carbonyl oxygen atoms from the amide moieties of two **L2** ligands. These four intramolecular N–H^**…**^O hydrogen bonding interactions play a crucial role in the stabilization of a figure-eight knot (Supplementary Fig. 5). In addition, the analogous Cp*Ir-based 4_1_ knot complex **3′** was separately constructed in a yield of 84% (Supplementary Fig. 6). In a word, the non-covalent interactions (NCIs), i.e., π–π stacking interactions, CH–π interactions and hydrogen bonding interactions, play synergetic roles in the formation of a figure-eight knot with four crossings.

### Self-assembly of binuclear trapezoidal macrocycle 4

In order to weaken the π–π stacking interactions between the pyridyl moieties and phenyl moieties in the trefoil knot structure, the longer ligand 2,5-dichloro-3,6-dihydroxy-1,4-benzoquinone (H_2_CA, **L3**) was deliberately chosen to build the edge unit **E3** [Cp*_2_Rh_2_(*μ*-CA)Cl_2_] upon formation of the macrocycle. By direct reactions of **E3** with ligand **L1**, the binuclear trapezoidal macrocycle **4** was obtained in a 95% yield rather than a tetranuclear ring as in complex **1** (Supplementary Fig. 13), and the structure of **4** was confirmed by NMR spectroscopy, ESI-MS and single-crystal X-ray diffraction analysis (Supplementary Fig. 7).

### Self-assembly of [2]catenane 5

**Fig. 8:**
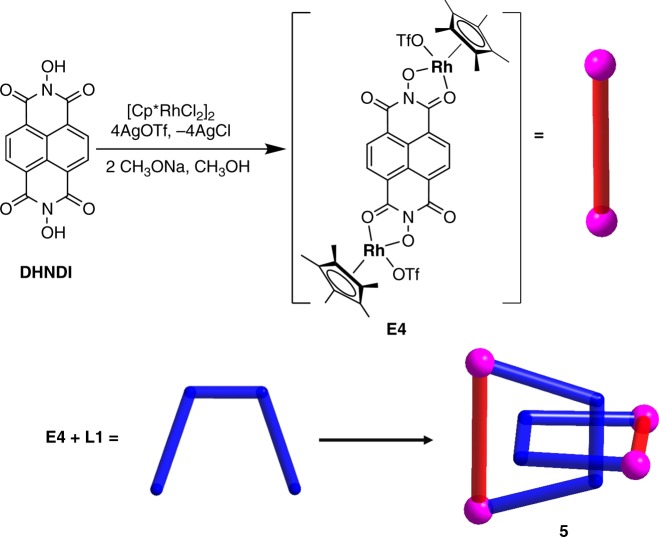
Synthesis of [2]catenane complex **5**. Schematic representation of the synthesis of **E4** (top) and stick model of **5** (below)

The parallel upper and bottom surfaces of **4**, as well as its cavity, prompted us to explore the synthesis of a molecular [2]catenane by employing an edge unit with a suitable electron-poor aromatic group. We speculated that favorable Donor-Acceptor stacking interactions between dinuclear edge unit **E4** and the flexible ligand **L1** may enable the self-assembly of a [2]catenane (Fig. 8). The length of the naphthalenediimide (NDI) edge unit is 11.9 Å^45^ (Rh–Rh nonbonding distance), which is large enough to allow the phenyl group of the flexible ligand **L1** to pass through.

As expected, the resulting [2]catenane complex **5** was formed in a yield of 93%, as revealed by NMR studies. The ^1^H NMR spectrum of **5** showed that the benzyl proton resonance was split into two broad signals (Supplementary Fig. 44) rather than the single resonance previously observed in macrocycle **4** (Supplementary Fig. 40), indicating the existence of a stable topological structure. Partial variable-temperature ^1^H NMR spectra of **5** showed that from 298 to 333 K, the split, broad signals of the NDI and benzyl groups merge into a single signal, suggesting that the [2]catenane topology limits the rotation of benzyl moieties on the ^1^H NMR timescale (Supplementary Fig. 49). The ^1^H DOSY NMR spectrum of **5** (Supplementary Fig. 46) showed that the aromatic and Cp* signals were associated with a single diffusion constant, suggesting that only one stoichiometry of assembly was formed. The ESI-MS data also indicated that complex **5** preserved its [2]catenane structure in solution: [**5**–OTf^–^]^+^ (*m/z* = 2687.20) (Supplementary Fig. 55). The proton signals remained unchanged over a wide concentration range (2.0–12.0 mM with respect to Cp*Rh; Supplementary Fig. 48), indicating that the Donor–Acceptor stacking interactions are strong enough to maintain the [2]catenane topology even in dilute solutions. This phenomenon contrasts with those observed in recent studies on [2]catenane structures^31,46,47^.

**Fig. 9:**
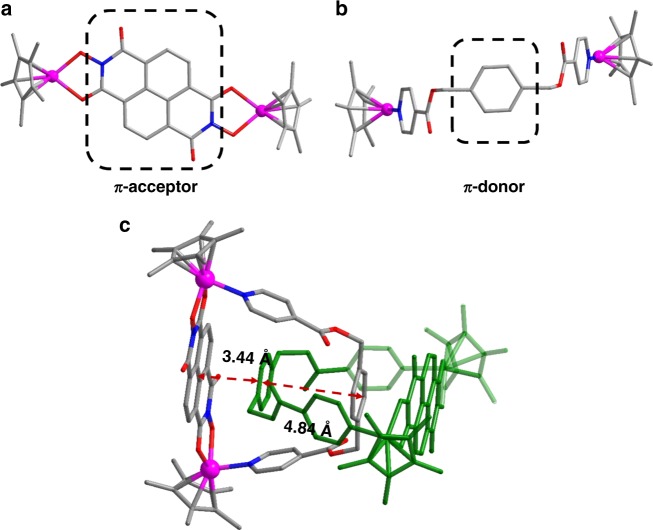
X-ray structure of **5** ([2]catenane). **a**,**b** depictions of the short and long arms (N, blue; O, red; C, gray; Rh, purple) (**c**) ball-and-stick representations. Counteranions are omitted for clarity

The X-ray structure of **5** confirmed its [2]catenane structure (Fig. 9), wherein two catenated trapezoids make up an inseparable ensemble. As expected, [2]catenane **5** is stabilized by strong π–π stacking interactions (3.44 Å, Fig. 9) between the NDI moieties of **E4** and the phenyl moieties of **L1**. In addition, there are no π–π stacking interactions between the phenyl moieties, the inter-ring distances being 4.84 Å (Fig. 9), much larger than the normal π–π stacking distance (~3.5 Å). In order to gain insight into the formation of **5**, DFT binding energy calculations were performed to study its intermolecular interactions (Supplementary Table 2). The energy of formation of the [2]catenane structure from two monomers of **4_3mo** (monomer like **4**, Supplementary Fig. 59) was evaluated to be −30.5 kcal/mol, while the contribution energy of π–π stacking was found to be −41.4 kcal/mol.

## Discussion

Transformations between monomeric macrocycles, molecular trefoil knots, and links are achieved by combining flexible ester ligand **L1** with different carefully chosen edge units through coordination-driven self-assembly. Above all, by employing amide ligand **L2**, a tetrahedral figure-eight knot comprising four crossings is realized, the solid-state structure of which shows that the molecule could display various forms, including a reduced representation with four crossings and a four-fold symmetry representation with eight crossings, merely by changing its conformation.

In conclusion, the π–π stacking, CH–π interactions, and hydrogen bonding interactions play synergistic roles in the formation of molecular trefoil knot and figure-eight knot. Our results thus demonstrate a controllable all-in-one approach for the creation of molecular knots and links through supramolecular interactions^1,2,25,48^, which we hope will further inspire the strategic design of topologically complex molecular architectures, molecular machines, and functional nanodevices.

## Methods

### Materials

All reagents and solvents were purchased from commercial sources and used as supplied unless otherwise mentioned. The starting materials [Cp*RhCl_2_]_2_ and [Cp*IrCl_2_]_2_ (Cp^*^ = *η*^5^-pentamethylcyclopentadienyl)^49^, BiBzIm (BiBzIm = 2,2´-bisbenzimidazole)^50^, and 2,7-dihydro-xybenzo[*lmn*][3,8]phenanthroline-1,3,6,8-(*2* *H,7* *H*)-tetraone (DHNDI)^43^ were prepared by literature methods.

### Characterization

NMR spectra were recorded on Bruker AVANCE I 400 and VANCE-DMX 500 spectrometers. Spectra were recorded at room temperature. Chemical shifts are reported relative to the solvent residual peaks (*δ*H = 3.31 (CD_3_OD)) or the solvent itself (*δ*C = 49.00 (CD_3_OD)). Coupling constants are expressed in Hertz. Elemental analyses were performed on an Elementar Vario EL III analyzer. IR spectra of the solid samples (KBr tablets) in the range 400–4000 cm^−1^ were recorded on a Nicolet AVATAR-360IR spectrometer. ESI-MS spectra were recorded on a UHR TOF LC/MS mass spectrometer using electrospray ionization.

### Synthesis of 2a and 2b

AgOTf (123.2 mg, 0.48 mmol) was added to a solution of [Cp*RhCl_2_]_2_ (74.0 mg, 0.12 mmol) in CH_3_OH (20 mL) at room temperature. The reaction mixture was stirred in the dark for 24 h and then filtered. BiBzIm (28.0 mg, 0.12 mmol) was added to the filtrate. The mixture was stirred at room temperature for 12 h to give a yellow solution. L1 (41.6 mg, 0.12 mmol) was then added. The mixture was stirred at room temperature for another 12 h to give a yellow solution. The solvent was concentrated to about 8 mL. Upon addition of diethyl ether, a yellow solid was precipitated and collected. The product was recrystallized from a CH_3_OH/diethyl ether mixture to afford a mixture of sheet-shaped crystals (2a) and needle-shaped crystals (2b).

Characterization data for **2a** and **2b**: 146.4 mg, total yield of crystals: 90%.

### 2a (monomeric tetranuclear macrocycle)

^1^H NMR (400 MHz, CD_3_OD, ppm, 2.0 mM, with respect to Cp*Rh): *δ* = 8.61 (d, *J* = 6.4 Hz, 8 H, pyridyl-αH), *δ* = 8.03 (q, *J* = 3.2 Hz, 8 H, BiBzIm-H), *δ* = 7.59 (d, *J* = 6.4 Hz, 8 H, pyridyl-βH), *δ* = 7.52 (q, *J* = 3.2 Hz, 8 H, BiBzIm-H), *δ* = 7.25 (s, 8 H, phenyl-H), *δ* = 5.30 (s, 4 H, benzyl-H), *δ* = 1.78 (s, 60 H, Cp*-H). Anal. Calcd for C_112_H_108_F_12_Rh_4_N_12_O_20_S_4_ (*M* = 2708.27): C, 49.64; H, 4.02; N, 6.20. Found: C, 49.45; H, 3.89, N, 6.12.

### 2b (trefoil knot)

^1^H NMR (400 MHz, CD_3_OD, ppm, 32.0 mM, with respect to Cp*Rh, saturated): *δ* = 8.71 (d, *J* = 6.0 Hz, 12 H, pyridyl-αH), *δ* = 8.19 (d, *J* = 8.8 Hz, 6 H, BiBzIm-H), *δ* = 8.12 (d, *J* = 8.8 Hz, 6 H, BiBzIm-H), *δ* = 7.52 (m, 6 H, BiBzIm-H), *δ* = 7.29 (d, *J* = 6.4 Hz, 12 H, pyridyl-βH), *δ* = 4.49 (d, *J* = 13.2 Hz, 6 H, phenyl-H), *δ* = 4.21 (s, 12 H, benzyl-H), *δ* = 2.40 (d, *J* = 13.2 Hz, 6 H, phenyl-H), *δ* = 1.75 (s, 90 H, Cp*-H). ^13^C{^1^H} (101 MHz, CD_3_OD, ppm): δ = 8.45 (Cp*), δ = 97.56 (d, *J* = 7.7 Hz, Cp*), 64.07, 116.09, 116.44, 123.20, 123.63, 123.71, 125.62, 129.72, 133.36, 138.04, 143.42, 153.84, 155.85, 162.03. IR (KBr disk, cm^−1^): *v* = 1734, 1606, 1451, 1415, 1378, 1355, 1278, 1224, 1158, 1123, 1058, 1031, 966, 911, 856, 809, 774, 764, 755, 697, 638, 573, 518, 495, 440. Anal. Calcd for C_168_H_162_F_18_Rh_6_ N_18_O_30_S_6_ (M = 4062.41): C, 49.64; H, 4.02; N, 6.20. Found: C, 49.55; H, 3.86, N, 6.13.

### Synthesis of 3 (figure-eight knot)

AgOTf (123.2 mg, 0.48 mmol) was added to a solution of [Cp*RhCl_2_]_2_ (72.0 mg, 0.12 mmol) in CH_3_OH (16 mL) at room temperature. The reaction mixture was stirred in the dark for 24 h and then filtered. BiBzIm (28.0 mg, 0.12 mmol) was added to the filtrate. The mixture was stirred at room temperature for 12 h to give a yellow solution. **L2** (41.6 mg, 0.12 mmol) was then added. The mixture was stirred at room temperature for another 12 h to give a yellow solution. The solvent was concentrated to about 7 mL. Upon the addition of diethyl ether, a yellow solid was precipitated and collected. The product was recrystallized from a CH_3_OH/diethyl ether mixture to afford a block-shaped crystals (3).

### Characterization data for 3 (4_1_ knot)

124.2 mg, yield 82%. ^1^H NMR (400 MHz, CD_3_OD, ppm, with respect to Cp*Rh): *δ* = 8.66 (d, *J* = 5.2 Hz, 8 H, pyridyl-αH), *δ* = 8.63 (d, *J* = 2.8 Hz, 8 H, pyridyl-αH), *δ* = 8.21 (d, *J* = 8.4 Hz, 4 H, BiBzIm-H), *δ* = 8.14 (t, *J* = 8.0 Hz, 4 H, BiBzIm-H), *δ* = 8.11 (t, *J* = 7.2 Hz, 4 H, BiBzIm-H), *δ* = 8.06 (d, *J* = 8.4 Hz, 4 H, BiBzIm-H),*δ* = 7.56 (t, *J* = 12.4 Hz, 4 H, BiBzIm-H), *δ* = 7.55 (d, *J* = 2.4 Hz, 4 H, BiBzIm-H), *δ* = 7.53 (s, 2 H, BiBzIm-H), *δ* = 7.51 (s, 2 H, BiBzIm-H), *δ* = 7.39 (m, *J* = 21.2 Hz, 4 H, BiBzIm-H), δ = 7.27 (d, *J* = 6.4 Hz, 8 H, pyridyl-βH), *δ* = 7.13 (d, *J* = 6.4 Hz, 8 H, pyridyl-βH), *δ* = 3.52 (dd, *J* = 14.8, 6 Hz, 8 H, phenyl-H), *δ* = 2.81 (dd, *J* = 14.8, 5.2 Hz, 8 H, phenyl-H), *δ* = 2.12 (dd, *J* = 17.2, 6 Hz, 8 H, benzyl-H), *δ* = 1.86 (dd, *J* = 16.8, 2.8 Hz, 8 H, benzyl-H), *δ* = 1.76 (s, 60 H, Cp*-H), δ = 1.68 (s, 60 H, Cp*-H). ^13^C{^1^H} (101 MHz, CD_3_OD, ppm): δ = 9.81 (Cp*), *δ* = 9.96 (Cp*), δ = 98.72(Cp*), *δ* = 98.79(Cp*), 43.47, 44.28, 117.26, 117.73, 118.46, 120.30, 123.47, 123.93, 124.87, 124.98, 125.08, 125.34, 125.56, 126.24, 128.68, 134.58, 136.26, 142.22, 144.53, 144.80, 145.04, 145.13, 154.50, 154.75, 157.27, 157.43, 164.43, 165.59. IR (KBr disk, cm^−1^): *v* = 1665, 1620, 1546, 1492, 1420, 1279, 1226, 1161, 1063, 1032, 855, 765, 640, 575, 518, 472. Anal. Calcd for C_224_H_224_F_24_Rh_8_ N_32_O_32_S_8_ (M = 5047.95): C, 53.30; H, 4.47; N, 1.66. Found: C, 53.32; H, 4.43, N, 1.69.

### Synthesis of 3′ (Cp*Ir-based figure-eight knot)

AgOTf (123.2 mg, 0.48 mmol) was added to a solution of [Cp*IrCl_2_]_2_ (96.0 mg, 0.12 mmol) in CH_3_OH (16 mL) at room temperature. The reaction mixture was stirred in the dark for 24 h and then filtered. BiBzIm (28.0 mg, 0.12 mmol) was added to the filtrate. The mixture was stirred at room temperature for 12 h to give a yellow solution. **L2** (41.6 mg, 0.12 mmol) was then added. The mixture was stirred at room temperature for another 12 h to give a yellow solution. The solvent was concentrated to about 8 mL. Upon the addition of diethyl ether, a yellow solid was precipitated and collected. The product was recrystallized from a CH_3_OH/diethyl ether mixture to afford a block-shaped crystal 3′. 145.2 mg, yield 84%.

### Synthesis of 5 ([2]catenane)

AgOTf (123.2 mg, 0.48 mmol) was added to a solution of [Cp*RhCl_2_]_2_ (74.0 mg, 0.12 mmol) in CH_3_OH (20 mL) at room temperature. The reaction mixture was stirred in the dark for 24 h and then filtered. 2,7-Dihydroxybenzo[*lmn*][3,8]phenanthroline-1,3,6,8-(2 H,7 H)-tetraone (DHNDI) (35.6 mg, 0.12 mmol) and NaOCH_3_ (12.8 mg, 0.24 mmol) were added to the filtrate. The mixture was stirred at room temperature for 24 h to give a dark red solution. **L1** (41.6 mg, 0.12 mmol) was added to the filtrate. The mixture was stirred at room temperature for another 24 h to give a dark red solution. The solvent was concentrated to about 8 mL. Upon addition of diethyl ether, a dark red solid precipitated and was collected. The product was recrystallized from a CH_3_OH/diethyl ether mixture to afford a dark red solid.

### Characterization data for 5

158.4 mg, yield 93%. ^1^H NMR (400 MHz, CD_3_OD, ppm): *δ* = 8.78 (d, *J* = 4.8 Hz, 8 H, pyridyl-αH), *δ* = 8.65 (br, 4 H, NDI-H), *δ* = 8.55 (br, 4 H, NDI-H), *δ* = 7.92 (d, *J* = 5.2 Hz, 8 H, pyridyl-βH), *δ* = 7.30 (s, 8 H, phenyl-H), *δ* = 5.60 (br, 4 H, benzyl-H), *δ* = 5.10 (br, 4 H, benzyl-H), *δ* = 1.81 (s, 60 H, Cp*-H). ^13^C{^1^H} (101 MHz, CD_3_OD, ppm): δ = 7.32 (Cp*), 95.39 (d, *J* = 9.2 Hz, Cp*), 21.62, 66.75, 123.75, 125.35, 128.91, 130.49, 131.43, 135.89, 140.37, 151.61, 162.91. IR (KBr disk, cm^−1^): *v* = 1731, 1627, 1585, 1552, 1502, 1458, 1418, 1380, 1266, 1225, 1158, 1123, 1059, 1031, 999, 982, 766, 751, 700, 639, 559, 518, 467. Anal. Calcd for C_112_H_100_F_12_Rh_4_N_8_O_32_S_4_ (*M* = 2836.14): C, 47.40; H, 3.55; N, 3.95. Found: C, 47.22; H, 3.60, N, 3.78. ESI-MS: *m*/*z* = 2687.20 (calcd for [M–OTf^–^]^+^ 2687.18).

## Supplementary information

Supplementary Information

## Data Availability

The X-ray crystallographic data reported in this Article have been deposited at the Cambridge Crystallographic Data Centre (CCDC), under deposition number CCDC 1888091 (**1**), 1870651 (**2a**), 1870652 (**2b**), 1870653 (**3**), 1870654 (**3′**), 1870655 (**4**), 1870656 (**5**). These data can be obtained free of charge from The Cambridge Crystallographic Data Centre via [www.ccdc.cam.ac.uk/data_request/cif]. The authors declare that all other data supporting the findings of this study are available within the paper and its supplementary information files.

## References

[1] Forgan RS, Sauvage JP, Stoddart JF (2011). Chemical topology: complex molecular knots, links, and entanglements. Chem. Rev..

[2] Sauvage JP (2017). From chemical topology to molecular machines (Nobel Lecture). Angew. Chem. Int. Ed..

[3] Fielden SDP, Leigh DA, Woltering SL (2017). Molecular knots. Angew. Chem. Int. Ed..

[4] Castelvecchi D (2017). The strange topology that is reshaping physics. Nat. News.

[5] Xu L, Wang YX, Chen LJ, Yang HB (2015). Construction of multiferrocenyl metallacycles and metallacages via coordination-driven self-assembly: from structure to functions. Chem. Soc. Rev..

[6] Clayton A, Vinograd J (1967). Circular dimer and catenate forms of mitochondrial DNA in human leukaemic leucocytes. Nature.

[7] Hudson B, Vinograd J (1967). Catenated circular DNA molecules in HeLa cell mitochondria. Nature.

[8] Liu LF, Depew RE, Wang JC (1976). Knotted single-stranded DNA rings: a novel topological isomer of circular single-stranded DNA formed by treatment with *Escherichia coli* ω protein. J. Mol. Biol..

[9] Richardson JS (1977). β-Sheet topology and the relatedness of proteins. Nature.

[10] Schappacher M, Deffieux A (2009). Imaging of catenated, figure-of-eight, and trefoil knot polymer rings. Angew. Chem. Int. Ed..

[11] Dietrich-Buchecker CO, Sauvage JP (1989). A synthetic molecular trefoil knot. Angew. Chem. Int. Ed..

[12] Nierengarten JF, Dietrich-Buchecker CO, Sauvage JP (1994). Synthesis of a doubly interlocked [2]-catenane. J. Am. Chem. Soc..

[13] Ponnuswamy. N, Cougnon FBL, Pantos GD, Sanders JKM (2014). Homochiral and meso figure eight knots and a solomon link. J. Am. Chem. Soc..

[14] Ayme JF (2011). A synthetic molecular pentafoil knot. Nat. Chem..

[15] Ayme JF (2012). Pentameric circular iron (II) double helicates and a molecular pentafoil knot. J. Am. Chem. Soc..

[16] Leigh DA, Pritchard RG, Stephens AJ (2014). A star of David catenane. Nat. Chem..

[17] Ayme JF, Beves JE, Campbell CJ, Leigh DA (2013). Template synthesis of molecular knots. Chem. Soc. Rev..

[18] Danon JJ (2017). Braiding a molecular knot with eight crossings. Science.

[19] Kim DH (2018). Coordination-driven self-assembly of a molecular knot comprising sixteen crossings. Angew. Chem. Int. Ed..

[20] Zhang Liang, Stephens Alexander J., Nussbaumer Alina L., Lemonnier Jean-François, Jurček Pia, Vitorica-Yrezabal Iñigo J., Leigh David A. (2018). Stereoselective synthesis of a composite knot with nine crossings. Nature Chemistry.

[21] Danon Jonathan J., Leigh David A., Pisano Simone, Valero Alberto, Vitorica-Yrezabal Iñigo J. (2018). A Six-Crossing Doubly Interlocked [2]Catenane with Twisted Rings, and a Molecular Granny Knot. Angewandte Chemie International Edition.

[22] Dietrich-Buchecker CO, Guilhem J, Pascard C, Sauvage JP (1990). Structure of a synthetic trefoil knot coordinated to two copper (I) centers. Angew. Chem., Int. Ed. Engl..

[23] Safarowsky O, Nieger M, Fröhlich R, Vögtle F (2000). A molecular knot with twelve amide groups-one-step synthesis, crystal structure, chirality. Angew. Chem., Int. Ed..

[24] Guo J, Mayers PC, Breault GA, Hunter CA (2010). Synthesis of a molecular trefoil knot by folding and closing on an octahedral coordination template. Nat. Chem..

[25] Barran PE (2011). Active-metal template synthesis of a molecular trefoil knot. Angew. Chem., Int. Ed..

[26] Ponnuswamy N, Cougnon FBL, Clough JM, Pantos GD, Sanders JKM (2012). Discovery of an organic trefoil knot. Science.

[27] Gil-Ramírez G (2016). Tying a molecular overhand knot of single handedness and asymmetric catalysis with the corresponding pseudo- *D*_3_-symmetric trefoil knot. J. Am. Chem. Soc..

[28] Zhang. L, Zhong JK, Whitehead GFS, Vitorica-Yrezabal IJ, Leigh DA (2018). Molecular trefoil knot from a trimeric circular helicate. J. Am. Chem. Soc..

[29] Huang Sheng-Li, Lin Yue-Jian, Li Zhen-Hua, Jin Guo-Xin (2014). Self-Assembly of Molecular Borromean Rings from Bimetallic Coordination Rectangles. Angewandte Chemie International Edition.

[30] Song YH (2016). Template-free synthesis of a molecular solomon link by two-component self-assembly. Angew. Chem. Int. Ed..

[31] Lee H (2015). Selective synthesis of ruthenium (II) metalla [2] catenane via solvent and guest-dependent self-assembly. J. Am. Chem. Soc..

[32] Oliveri CG, Ulmann PA, Wiester MJ, Mirkin CA (2008). Heteroligated supramolecular coordination complexes formed via the halide-induced ligand rearrangement reaction. Acc. Chem. Res..

[33] Fujita M, Tominaga M, Hori A, Therrien B (2005). Coordination assemblies from a Pd(II)-cornered square complex. Acc. Chem. Res..

[34] Pluth MD, Bergman RG, Raymond KN (2009). Proton-mediated chemistry and catalysis in a self- assembled supramolecular host. Acc. Chem. Res..

[35] Lu Y, Zhang HN, Jin GX (2018). Molecular Borromean rings based on half-sandwich organometallic rectangles. Acc. Chem. Res..

[36] Yoshizawa M, Klosterman JK, Fujita M (2009). Functional molecular flasks: new properties and reactions within discrete, self-assembled hosts. Angew. Chem. Int Ed..

[37] Cook TR, Stang PJ (2015). Recent developments in the preparation and chemistry of metallacycles and metallacages via coordination. Chem. Rev..

[38] Alexander JW, Briggs GB (1926). On types of knotted curves. Ann. Math..

[39] Cougnon FBL, Au-Yeung HY, Pantoş DG, Sanders JKM (2011). Exploring the formation pathways of donor-acceptor catenanes in aqueous dynamic combinatorial libraries. J. Am. Chem. Soc..

[40] Cougnon FBL, Ponnuswamy N, Jenkins NA, Pantoş DG, Sanders JKM (2012). Structural parameters governing the dynamic combinatorial synthesis of catenanes in water. J. Am. Chem. Soc..

[41] Ronson TK, Roberts DA, Black SP, Nitschke JR (2015). Stacking interactions drive selective self- assembly and self-sorting of pyrene-based M^II^_4_L_6_ architectures. J. Am. Chem. Soc..

[42] Kim T (2016). Selective synthesis of molecular borromean rings: engineering of supramolecular topology via coordination-driven self-assembly. J. Am. Chem. Soc..

[43] Lu Y (2017). Molecular Borromean rings based on dihalogenated ligands. Chem.

[44] Huang SL, Lin YJ, Hor TSA, Jin GX (2013). Cp*Rh-based heterometallic metallarectangles: size- dependent Borromean link structures and catalytic acyl transfer. J. Am. Chem. Soc..

[45] Zhang L (2017). Stacking interactions induced selective conformation of discrete aromatic arrays and Borromean rings. J. Am. Chem. Soc..

[46] Liu NF, Huang SL, Liu XG, Luo HK, Andy Hor TS (2017). Self-assembled [2]catenane in trapezoidal metallacycles with [Cp*Ir]-corners. Chem. Commun..

[47] Jo JH (2017). Coordination-driven self-assembly using ditopic pyridyl–pyrazolyl donor and p-Cymene Ru(II) cceptors: [2]Catenane synthesis and anticancer activities. Inorg. Chem..

[48] Prakasam T (2013). ` Simultaneous self-assembly of a [2]Catenane, a trefoil knot, and a solomon link from a simple pair of ligands. Angew. Chem. Int. Ed..

[49] White C, Yates A, Maitlis PM (1992). *η*^5^-Pentamethylcyclopentadienyl) rhodium and -iridium compounds. Inorg. Synth..

[50] Wu T, Weng LH, Jin GX (2012). Sunlight induced cycloaddition and host–guest property of self-assembled organometallic macrocycles based on a versatile building block. Chem. Comm..

