# Supplementary material for: Coordination-driven self-assembly of a molecular figure-eight knot and other topologically complex architectures
Source: Nat Commun. 2019 May 3;10:2057. doi: 10.1038/s41467-019-10075-6 (PMC6499799; doi:10.1038/s41467-019-10075-6)
Supplement: Supplementary file 1 — Supplementary Information [file 41467_2019_10075_MOESM1_ESM.pdf]

Supplementary information for

Coordination-Driven Self-Assembly of a Molecular Figure-Eight Knot and Other Topologically Complex Architectures

L.L. Dang, Z.B. Sun, W.L. Shan, Y.J. Lin, Z.H. Li and G.X. Jin*

1. Supplementary Figures: Single-crystal X-ray structure of 1, 2b, 3, 3′ and 4

1.1. Single-crystal X-ray structure of 1

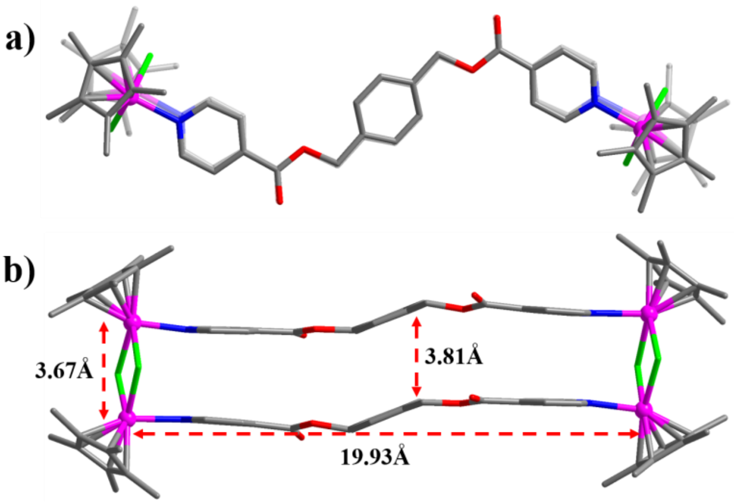

Supplementary Figure 1. Single-crystal X-ray structure of **1**, top view (a) and front view (b). Counteranions and hydrogen atoms are omitted for clarity (N, blue; O, red; C, gray; Rh, purple; Cl, green).

1.2. Single-crystal X-ray structure of 2b

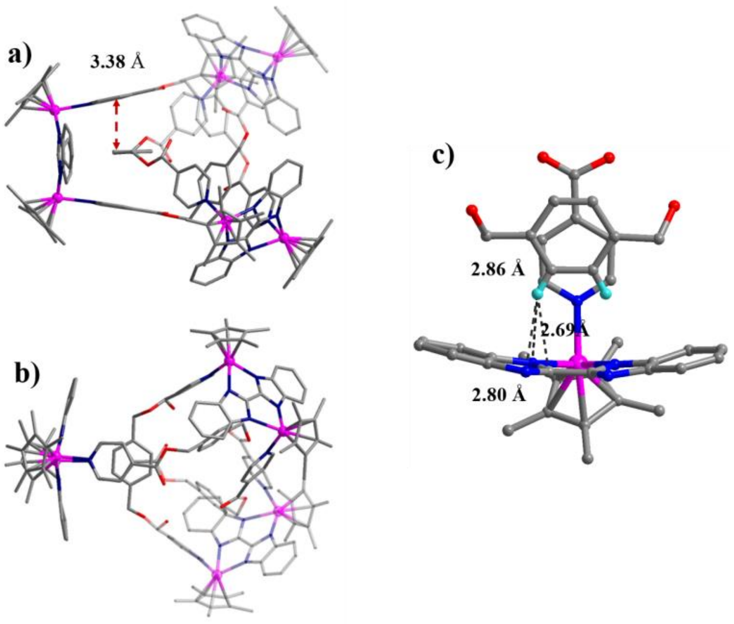

Supplementary Figure 2. Single-crystal X-ray structure of **2b**, π-π interactions between the pyridine moiety and benzene moiety, front view (**a**), top view (**b**). Distances from proton of phenyl moiety to BiBzIm (**c**). Counteranions and other hydrogen atoms are omitted for clarity (N, blue; O, red; C, gray; Rh, purple; H, light blue).

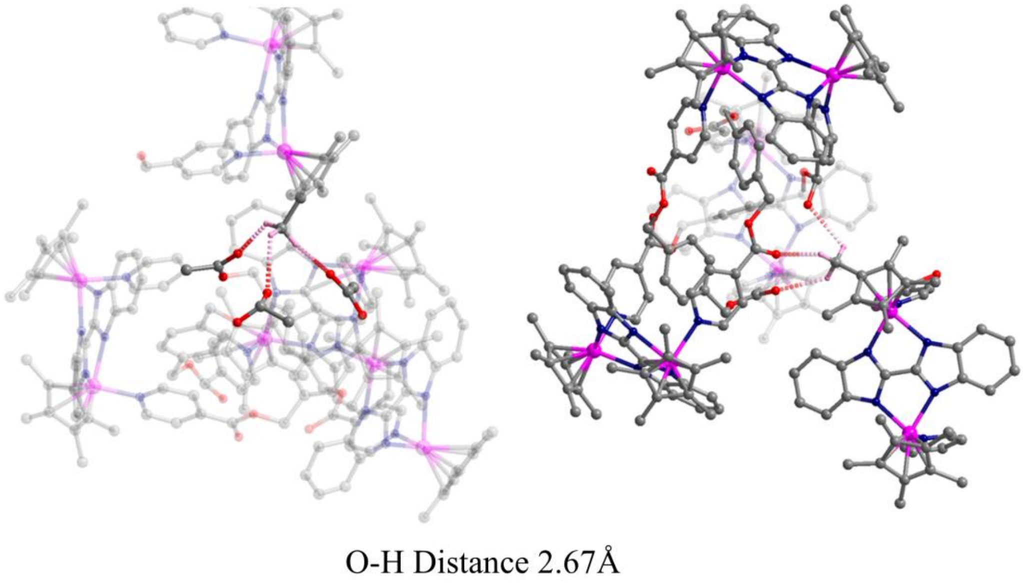

Supplementary Figure 3. Single-crystal X-ray structure of **2b**, hydrogen bonds between O of ester moieties and Cp* protons. Counteranions and other hydrogen atoms are omitted for clarity (N, blue; O, red; C, gray; Rh, purple; H, light pink).

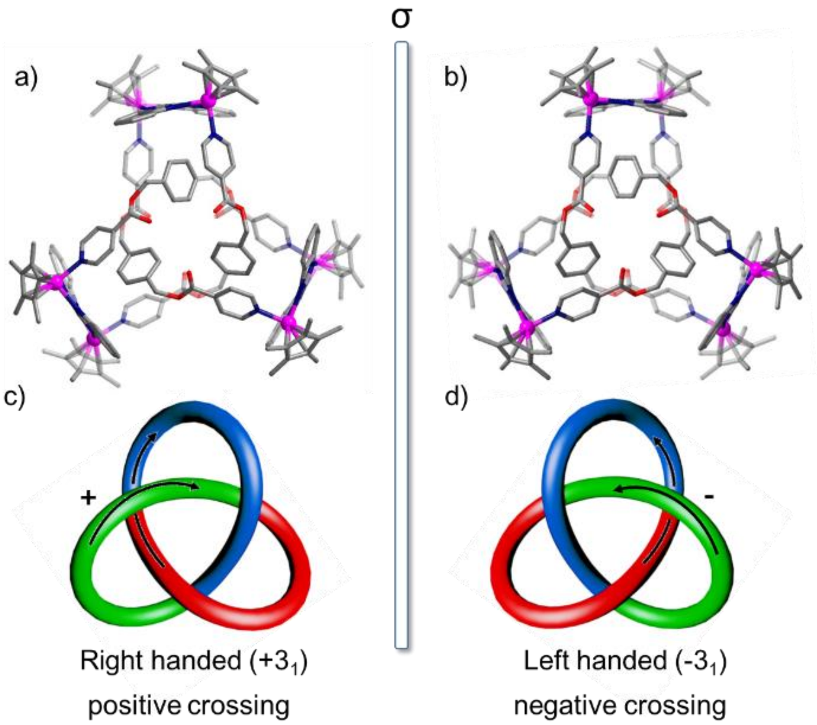

Supplementary Figure 4. Two mirror symmetric single-crystal X-ray structures of **2b** (**a**, **b**). Simplified structures in the form of bending string (**c**, **d**).

1.3. Single-crystal X-ray structure of 3

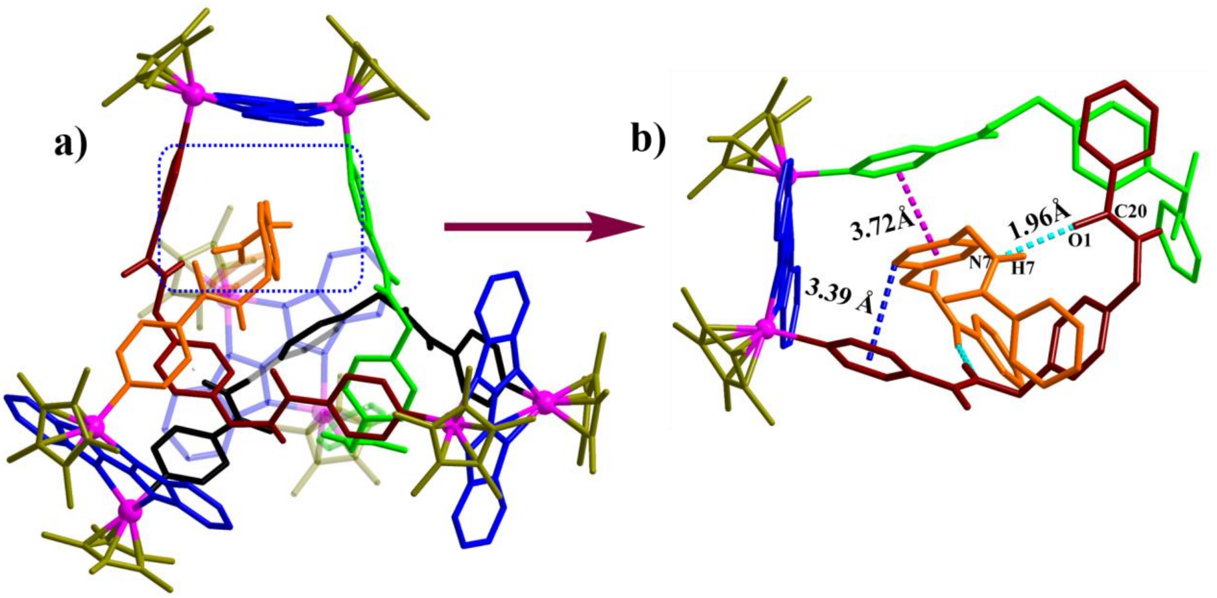

Supplementary Figure 5. Single-crystal X-ray structure of **3 (**with respect to Cp*Rh**)** (bottom view), π-π interactions and CH-π interactions between the pyridine moiety and benzene moiety, N-H···O hydrogen bonds between two **L2** ligands and other hydrogen atoms are omitted for clarity.

1.4. Single-crystal X-ray structure of 3′

The data obtained for **3′** is almost the same as **3**, except with iridium centers instead of rhodium centers.

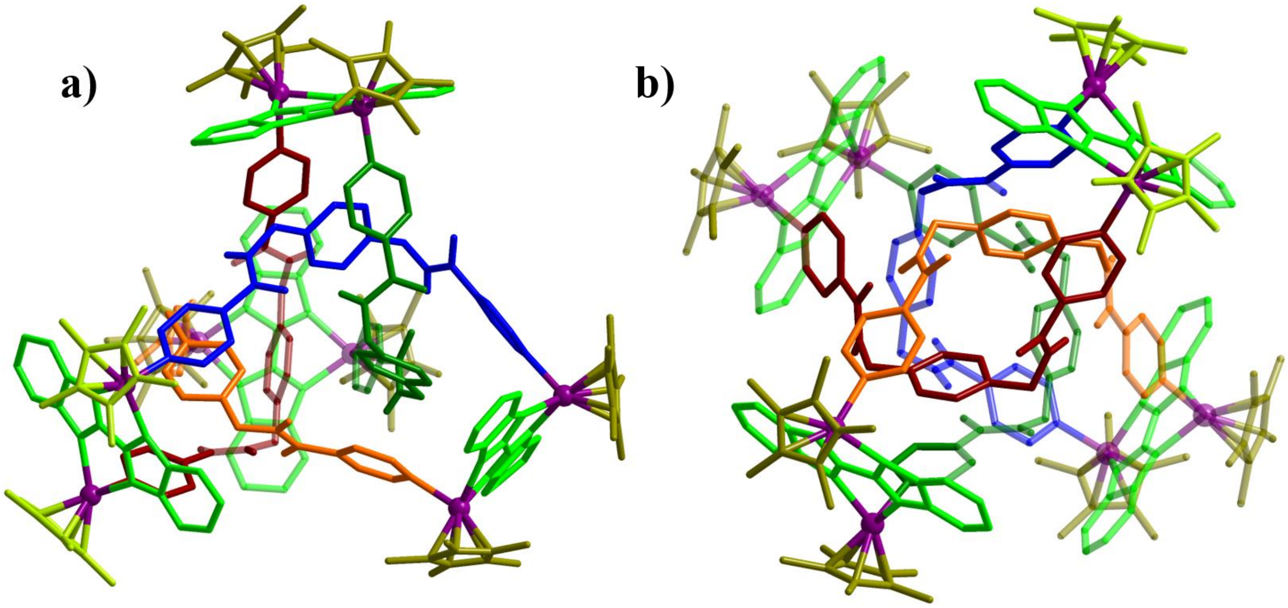

Supplementary Figure 6. Single-crystal X-ray structure of Cp*Ir-Based figure eight knot 3′. The reduced representation with four crossings (**a**) and the 4-fold symmetries representation (**b**) in stick model of **3**.

1.5. Single-crystal X-ray structure of 4

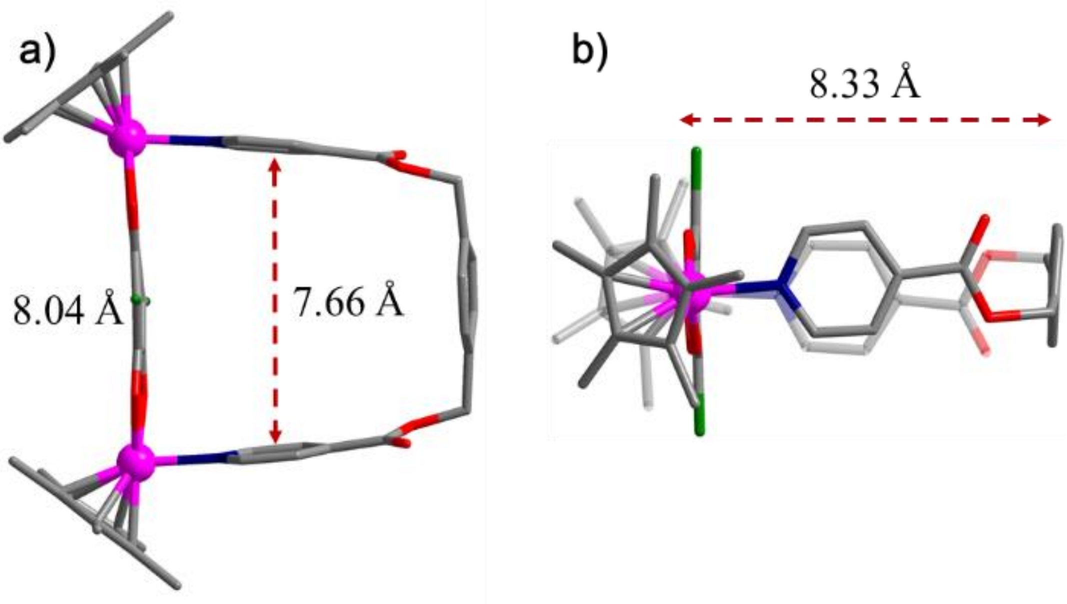

Supplementary Figure 7. Single-crystal X-ray structure of **4**: side view (**a**), and top view (**b**). Counteranions and hydrogen atoms are omitted for clarity. (N, blue; O, red; C, gray; Rh, purple; Cl, green).

2. Supplementary Methods: Synthesis of Ligand L1 and L2

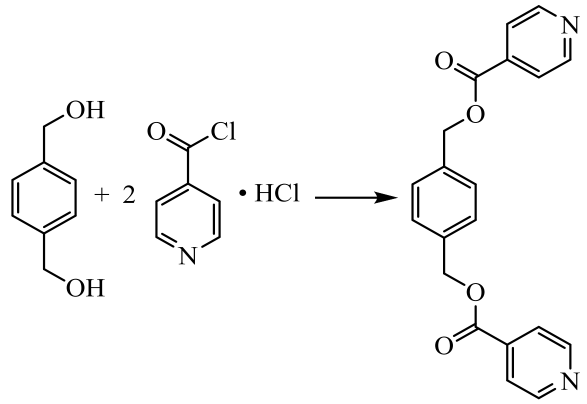

Supplementary Figure 8. Synthetic route to **L1** ligand.

Synthesis of 1,4-phenylenebis(methylene) diisonicotinate: A mixture of 2,5-bis(hydroxymethyl)benzene (0.4145 g, 3 mmol), isonicotinoyl chloride hydrochloride (1.068 g, 6 mmol), DMAP (cat., 30 mg) was dissolved in 30mL anhydrous DCM and stirred at r.t. for 1 h under nitrogen. Afterwards, Et_3N (0.6071 g, 6 mmol) was added and further stirred for 12 h. White precipitate was collected by filtration and washed by water (2×5mL), DCM (2×5mL), Et_2O (2×5mL) and dried. The yields: 1.212 g, 58%. ^{1}H NMR ($CDCl_3$, 400 MHz) δ: 8.78 (d, J = 4.8 Hz, 4H), 7.87 (d, J = 4.4 Hz, 4H), 7.49 (s, 4H), 5.41 (s, 4H); elemental analysis calcd (%) for $C_{18}H_{13}N_3$: C: 68.96, H: 4.63, N: 8.04; found C: 68.88, H: 4.65, N: 7.99.

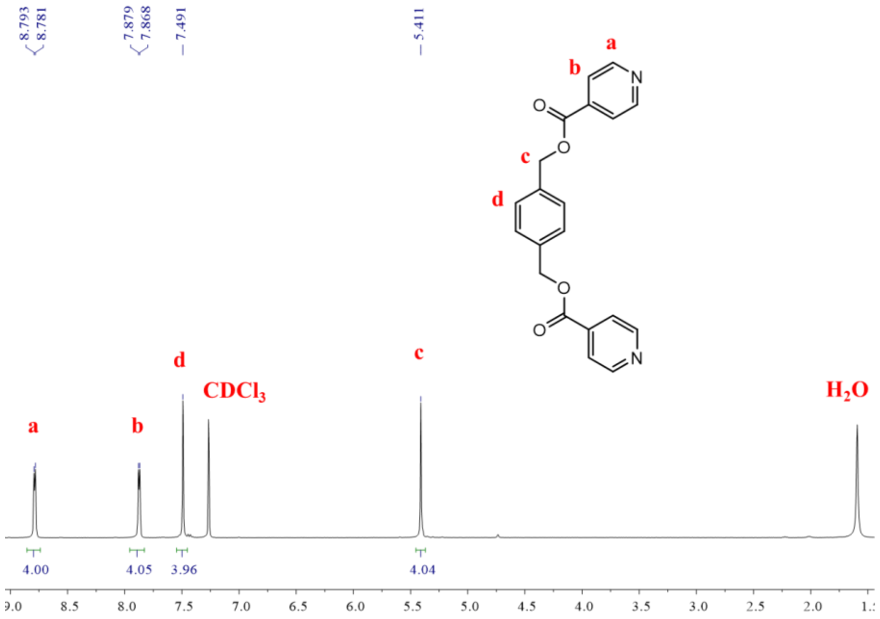

Supplementary Figure 9. ^{1}H NMR spectrum of 1,4-phenylenebis(methylene) diisonicotinate

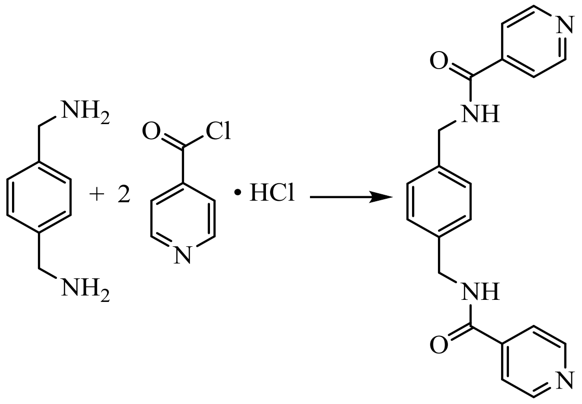

Supplementary Figure 10. Synthetic route to **L2** ligand.

Synthesis of N,N′-[1,4-phenylenebis(methylene)]bis-4-pyridinecarboxamide: A mixture of 1,4-Benzenedimethanamine (0.5404 g, 4 mmol) and isonicotinoyl chloride hydrochloride(1.4596 g, 8.2 mmol) was dissolved in 30mL anhydrous DCM and stirred at r.t. for 1h under nitrogen. Afterwards, Et_3N (1.25 mL, 9 mmol) was added dropwise and further stirred for 30 h. A white solid was obtained after quenching the reaction with aqueous bicarbonate solution. This was collected by filtration, washed with dichloromethane, ethanol, diethyl ether and dried. The yields: 748 mg, 54%. ^1H NMR ($(CD_3)_2SO$, 400 MHz) δ: 9.33 (br s, J = 0.8 Hz, 2H), 8.72 (d, J = 4.4 Hz, 4H), 7.49 (d, J = 4.4 Hz, 4H), 7.29 (s, 4H), 4.46 (d, J = 5.6 Hz, 4H); elemental analysis calcd (%) for $C_{20}H_{18}N_4O_2$: C: 69.35, H: 5.24, N: 16.17; Found C: 69.31, H: 5.26, N: 16.20.

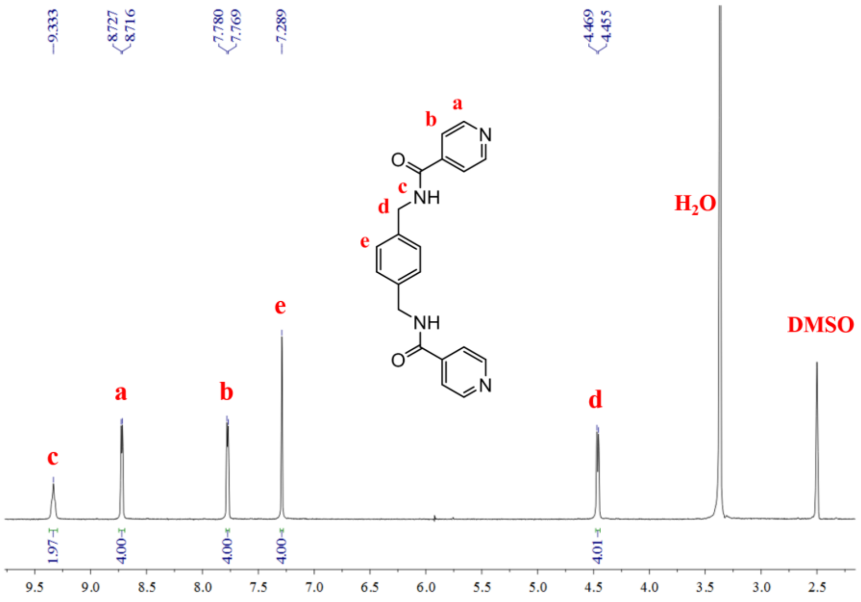

Supplementary Figure 11. ^1H NMR spectrum of N,N’-[1,4-phenyl enebis(methylene)] bis-4-pyridine carbox-amide.

3. Supplementary Methods: Synthesis of complexes 1 and 4

3.1 Synthesis of complex 1.

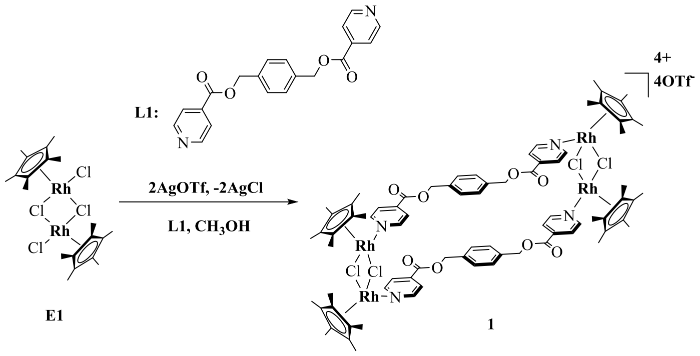

Supplementary Figure 12. Synthesis of tetranuclear complex 1.

AgOTf (15.4 mg, 0.06 mmol) was added to a solution of $[Cp^*RhCl_2]_2$ (18.5 mg, 0.03 mmol) in CH_3OH (5 mL) at room temperature. The reaction mixture was stirred in the dark for 24 h and then filtered. **L1** (10.4 mg, 0.03 mmol) was added to the filtrate. The mixture was stirred at room temperature for 24 h to give an orange solution. The solvent was then concentrated to about 2 mL. Upon the addition of diethyl ether, an orange solid precipitated and was collected. The product was recrystallized from a CH_3OH/diethyl ether mixture to afford an orange solid. 32.9 mg, yield 92%. IR (KBr disk, cm^{-1}): ν = 1734, 1617, 1453, 1418, 1378, 1281, 1225, 1159, 1127, 1059, 857, 834, 765, 699, 574, 518, 446. Anal. Calcd for $C_{84}H_{92}F_{12}Rh_4N_4O_{20}S_4Cl_4$ (M = 2384.00): C, 42.26; H, 3.88; N, 2.35. Found: C, 42.12; H, 3.79, N, 2.38. ESI-MS: m/z = 2235.04 (calcd for $[M - OTf^-]^+$ 2235.04).

3.2 Synthesis of complex 4.

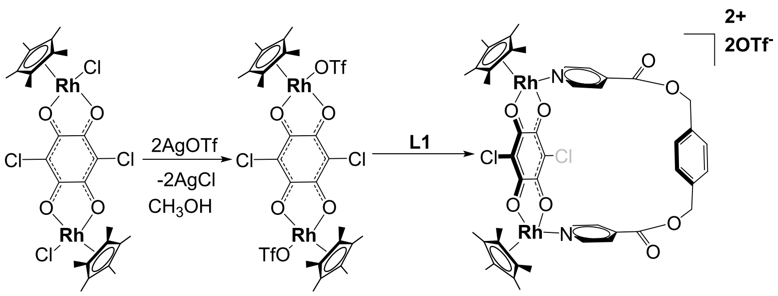

E3 4

Supplementary Figure 13. Synthesis of dinuclear complex **4**.

AgOTf (15.4 mg, 0.06 mmol) was added to a solution of [Cp*$_2$Rh$_2$(μ-CA)Cl$_2$] (22.6 mg, 0.03 mmol) in CH_3OH (5 mL) at room temperature. The reaction mixture was stirred in the dark for 24 h and then filtered. **L1** (10.4 mg, 0.03 mmol) was added to the filtrate. The mixture was stirred at room temperature for 24 h to give a dark green solution. The solvent was concentrated to about 2 mL. Upon the addition of diethyl ether, a dark green solid precipitated and was collected. The product was recrystallized from a CH_3OH/diethyl ether mixture to afford a dark green solid. 37.9 mg, yield 95%. IR (KBr disk, cm^{-1}): v = 1734, 1618, 1494, 1419, 1374, 1280, 1225, 1159, 1122, 1059, 1031, 861, 765, 698, 575, 517, 500. Anal. Calcd for $C_{48}H_{46}F_6Rh_2N_2O_{14}S_2Cl_2$ (M = 1327.98): C, 43.36; H, 3.49; N, 2.11. Found: C, 43.52; H, 3.30, N, 2.03. ESI-MS: m/z = 1179.03 (calcd for [M – OTf$^-$]$^+$ 1179.03), m/z = 515.04 (calcd for [M − 2OTf$^-$]$^{2+}$ 515.04).

4. Supplementary Figures: NMR spectra

4.1 NMR spectrum of 1

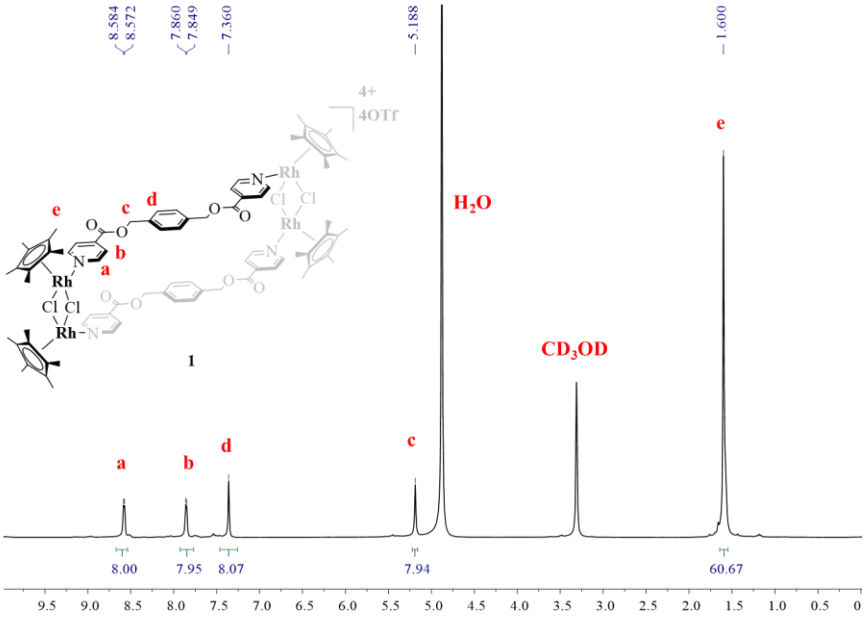

Supplementary Figure 14. ^{1}H NMR (400 MHz, CD$_3$OD, ppm) for **1** (20.0 mM, with respect to Cp*Rh)**.**

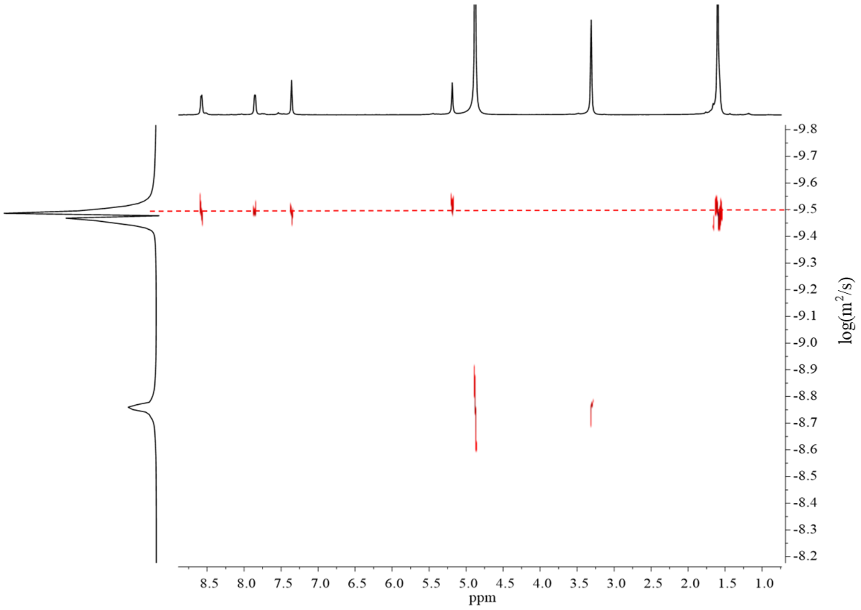

Supplementary Figure 15. ^{1}H DOSY NMR (400 MHz, CD$_3$OD, ppm) for **1** (20.0 mM, with respect to Cp*Rh)**.**

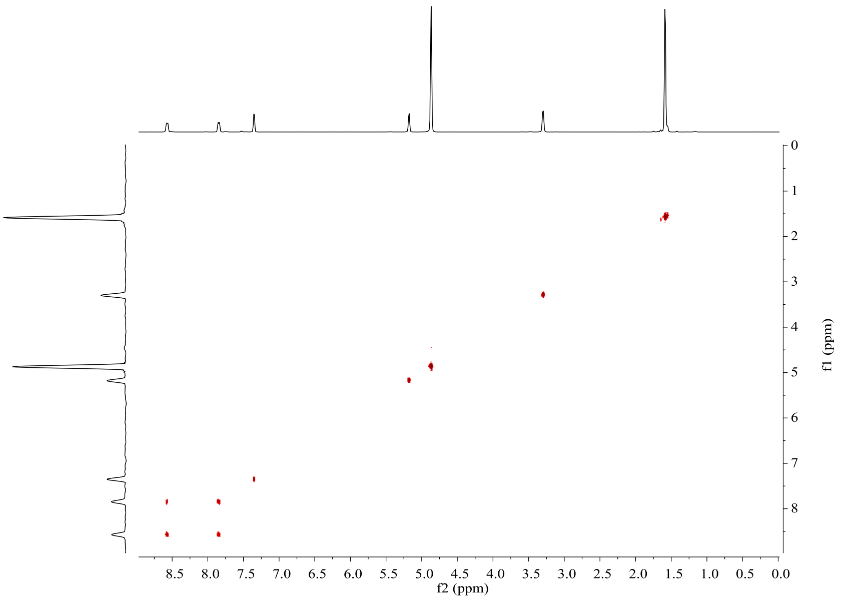

Supplementary Figure 16. ^{1}H COSY NMR (400 MHz, CD_3OD, ppm) for **1** (20.0 mM, with respect to Cp*Rh)**.**

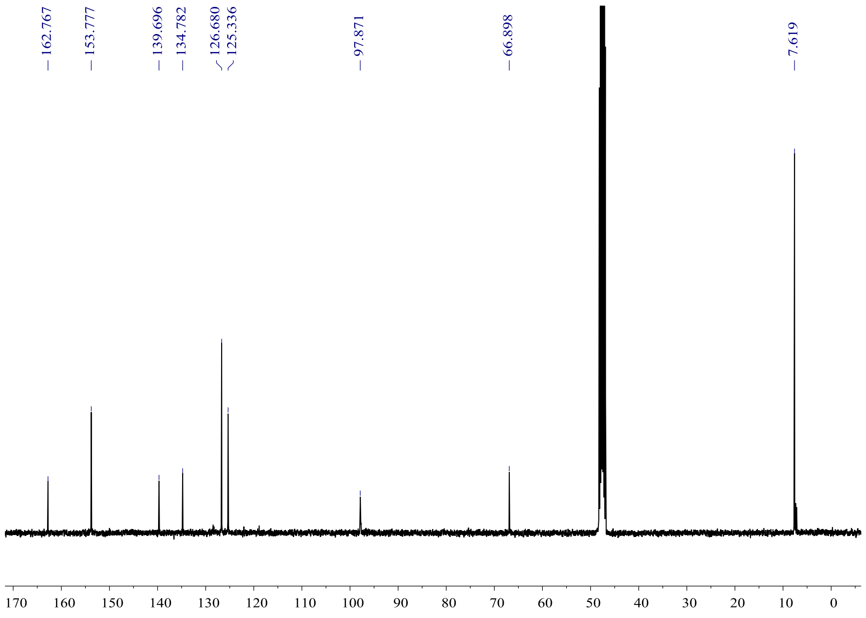

Supplementary Figure 17. ^{13}C{^{1}H} NMR (101 MHz, CD_3OD, ppm) for **1** (20.0 mM, with respect to Cp*Rh)**.**

4.2 NMR spectrum for 2a

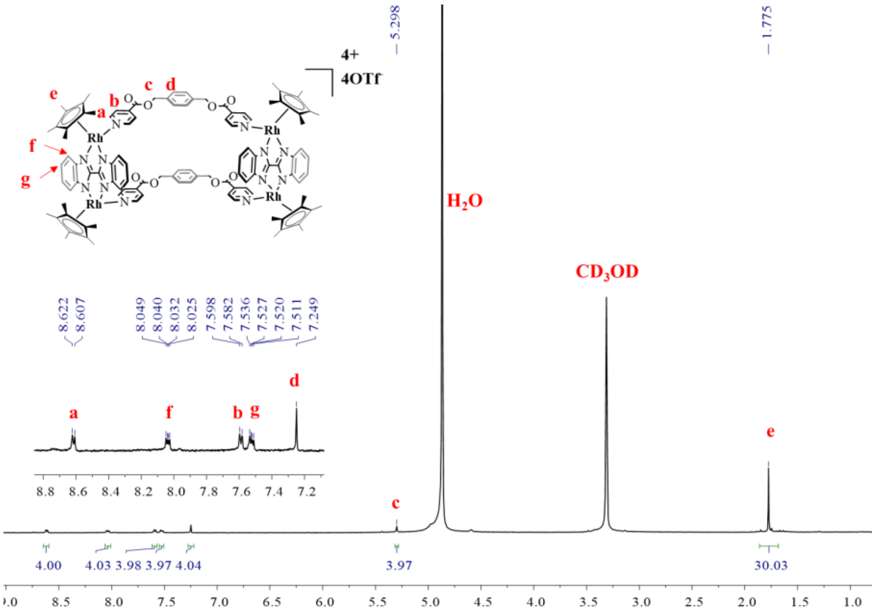

Supplementary Figure 18. ^{1}H NMR (400 MHz, CD_3OD, ppm) for **2a** (2.0 mM, with respect to Cp*Rh)**.**

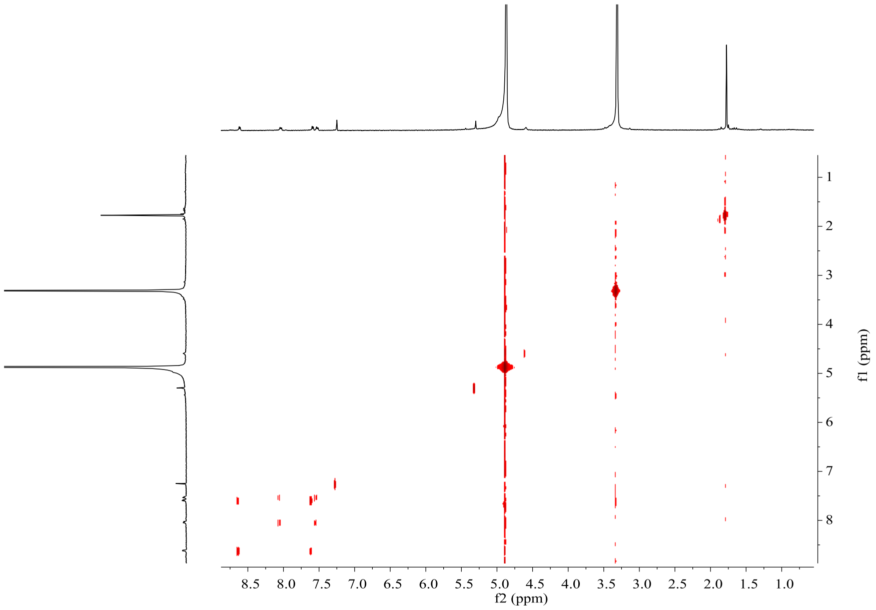

Supplementary Figure 19. ^{1}H COSY NMR (400 MHz, CD_3OD, ppm) for **2a** (2.0 mM, with respect to Cp*Rh)**.**

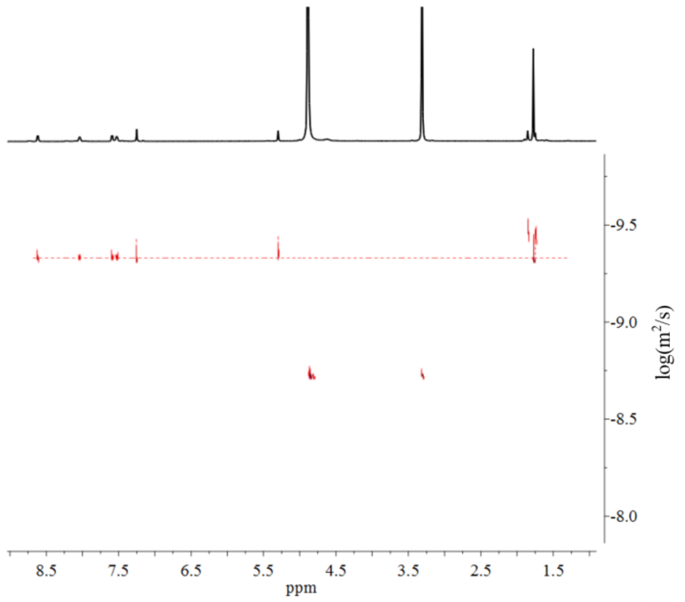

Supplementary Figure 20. ^{1}H DOSY NMR (500 MHz, CD_3OD, ppm) for **2a** (2.0 mM, with respect to Cp*Rh) Diffusion coefficient: 4.7×10^{-10} m^2s^{-1}**.**

4.3 NMR spectrum for 2b

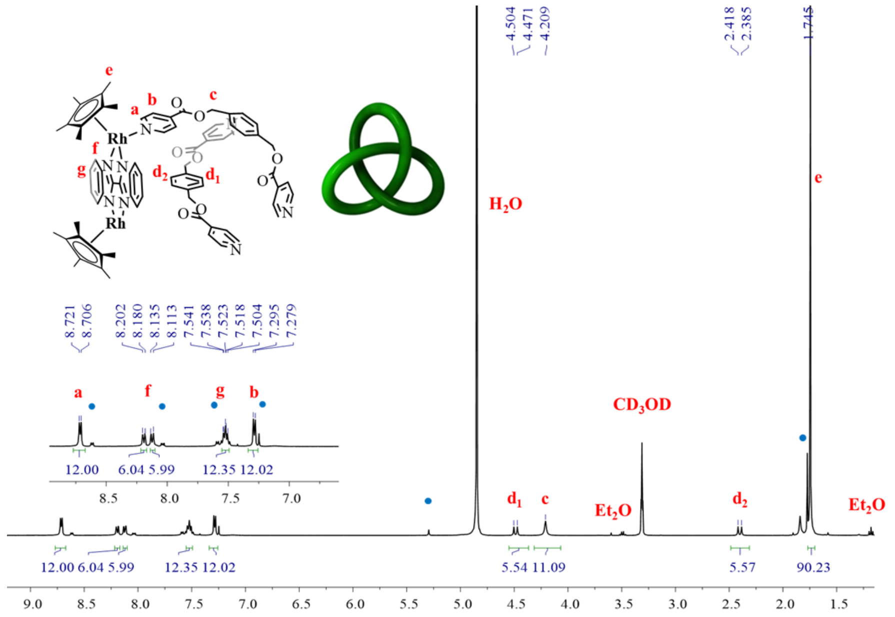

Supplementary Figure 21. ^{1}H NMR (400 MHz, CD_3OD, ppm) for **2b.** (32.0 mM, with respect to Cp*Rh)

The small peaks marked with red dots are belong to **2a**.

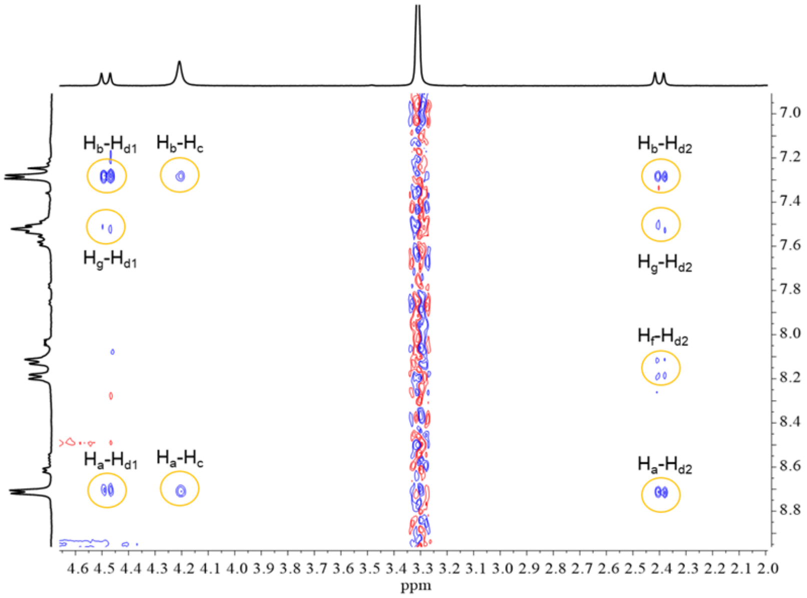

Supplementary Figure 22. ROESY NMR (500 MHz, CD_3OD, ppm) for **2b**. (32.0 mM, with respect to Cp*Rh)

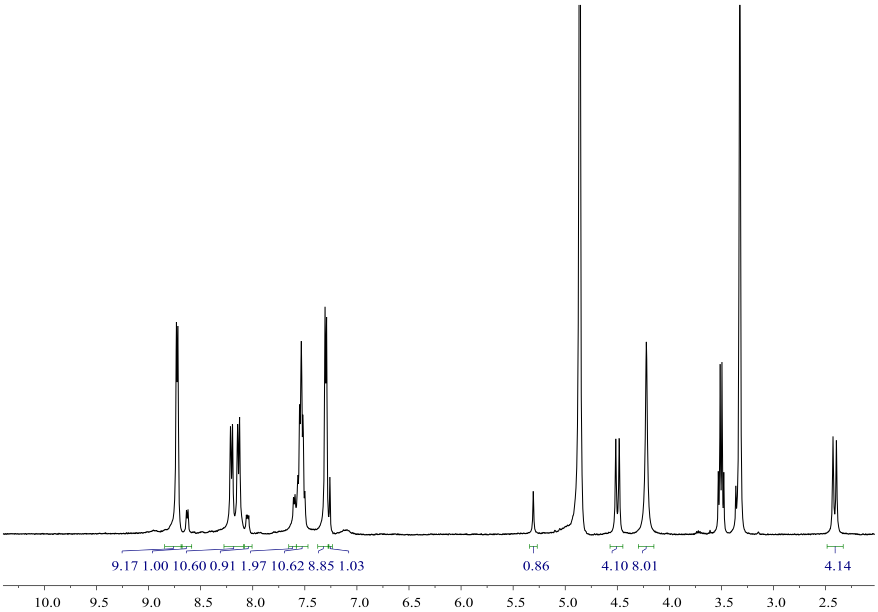

Supplementary Figure 23. Mass% of **2a** and **2b** in saturated solution of **2.**

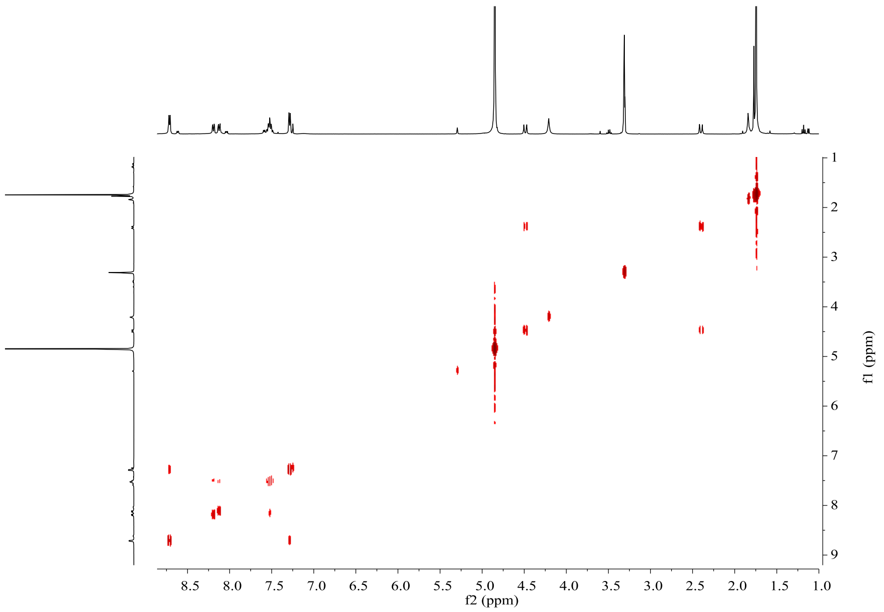

Supplementary Figure 24. ^{1}H COSY NMR (400 MHz, CD_3OD, ppm) for **2b**. (32.0 mM, with respect to Cp*Rh) The 1H NMR signals at 4.49 ppm and 2.40 ppm have a coupling interaction and they belong to phenyl protons which have a signal at 7.20 ppm in **2a**. Large upfield shifts of phenyl protons indicate tight π-π stacking between phenyl and pyridyl groups, which contributes to the formation of trefoil knot **2b** and the stable structure of **2b** results in the splitting of phenyl protons.

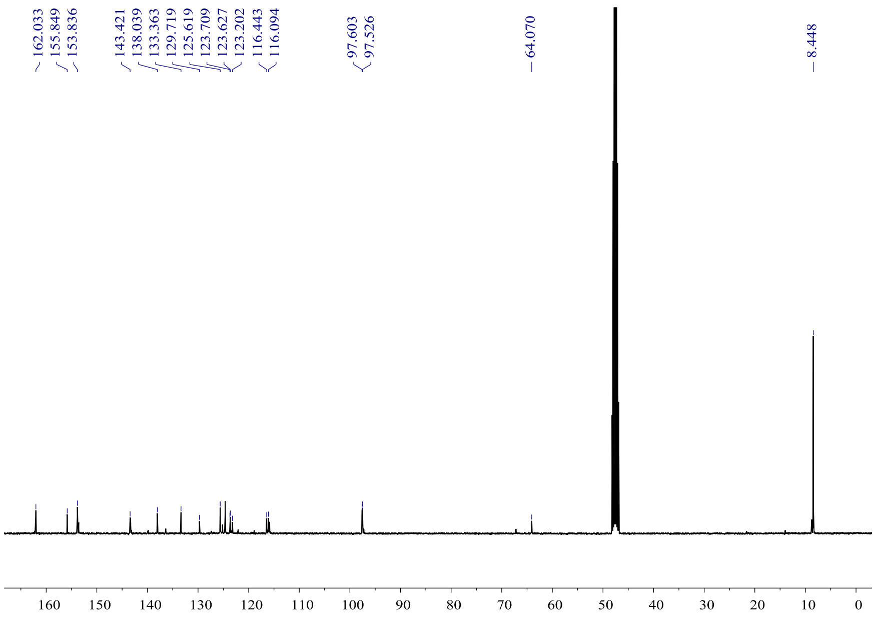

Supplementary Figure 25. $^{13}C\{^{1}H\}$ NMR (101 MHz, CD_3OD, ppm) for **2b**. (32.0 mM, with respect to Cp*Rh)

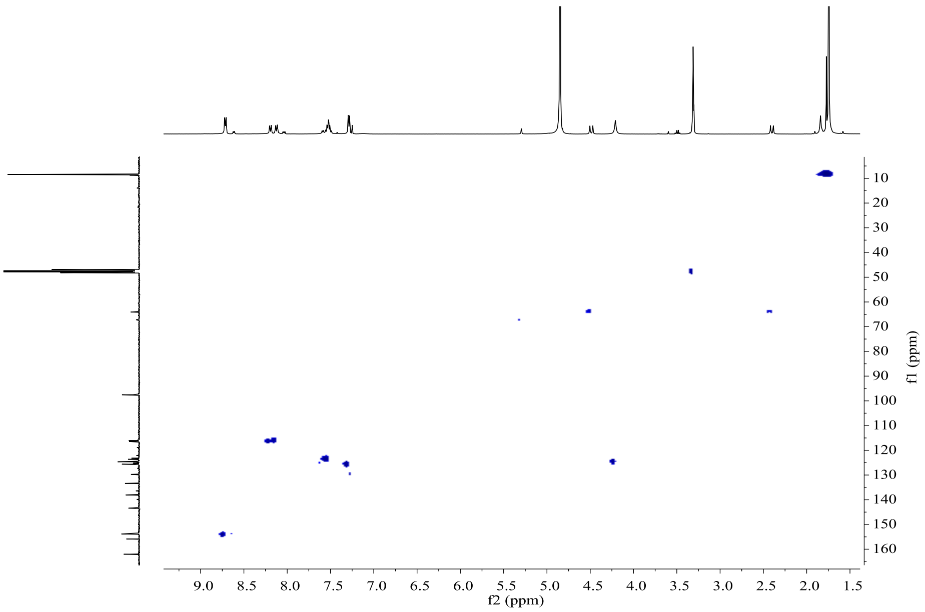

Supplementary Figure 26. ^{1}H -^{13}C HSQC NMR (400 MHz, CD_3OD, ppm) for **2b** (32.0 mM, with respect to Cp*Rh).

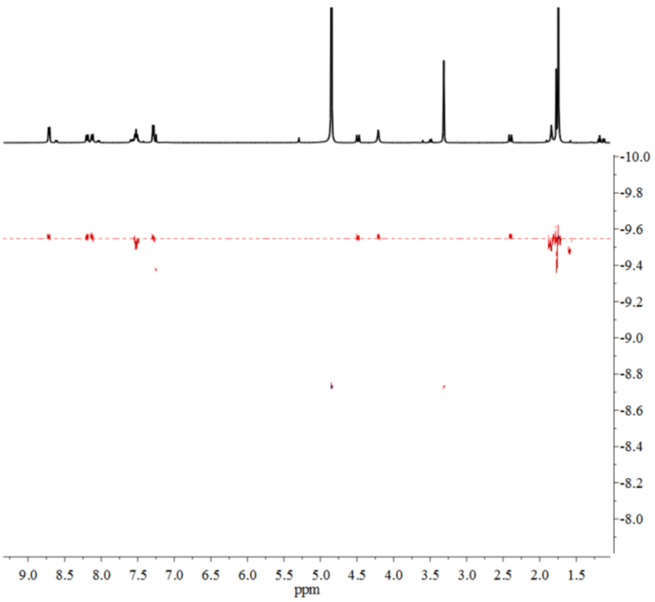

Supplementary Figure 27. ^{1}H DOSY NMR (500 MHz, CD_3OD, ppm) for **2b** (32.0 mM, with respect to Cp*Rh) Diffusion coefficient: 2.8×10^{-10} m^2s^{-1}**.**

4.4 NMR spectrum for 2a+2b

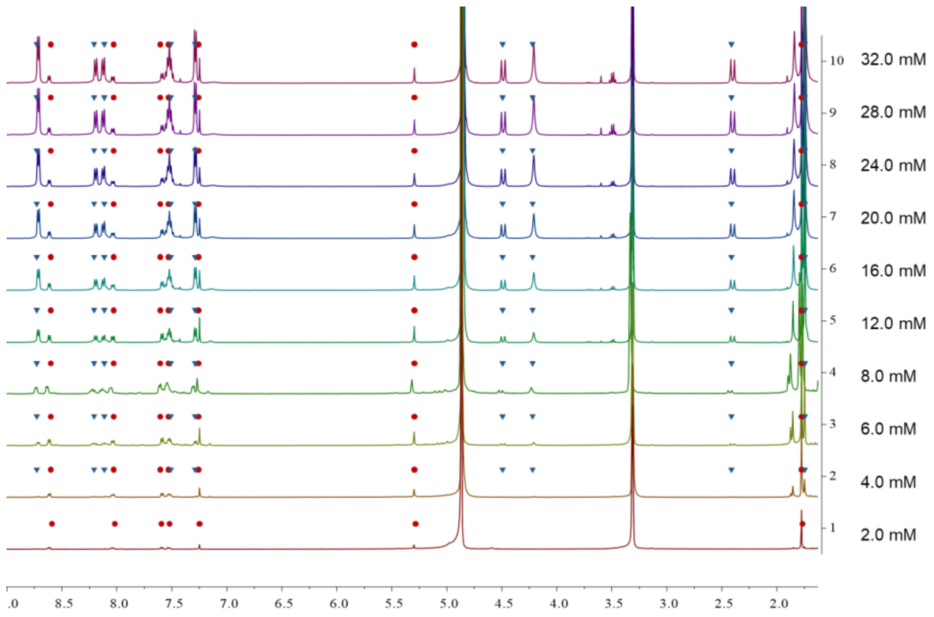

Supplementary Figure 28. ^{1}H NMR (400 MHz, CD_3OD, ppm) for **2a+2b** (2.0 mM to 32.0 mM, with respect to Cp*Rh).

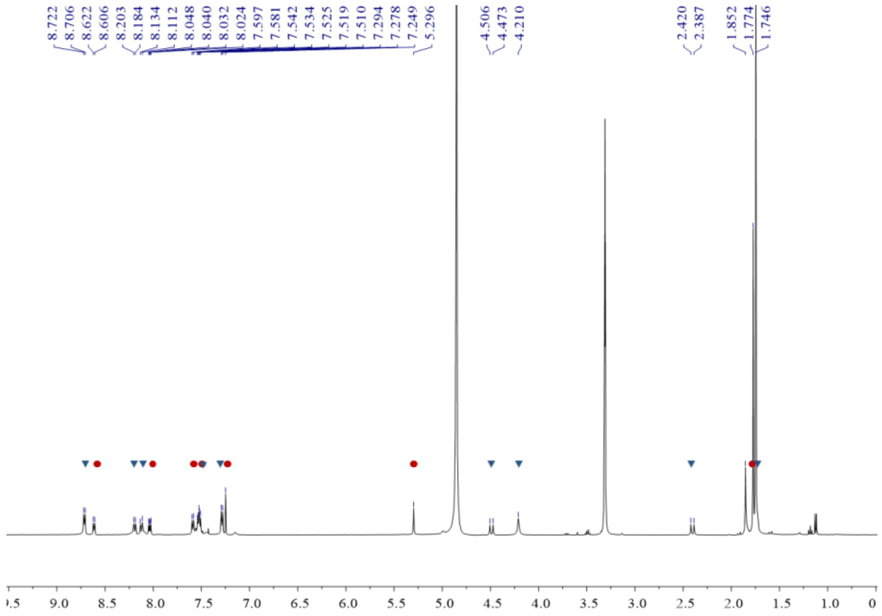

Supplementary Figure 29. ^{1}H NMR (400 MHz, CD_3OD, ppm) for **2a+2b** (16.0mM, Red circle for **2a**, blue triangle for **2b**).

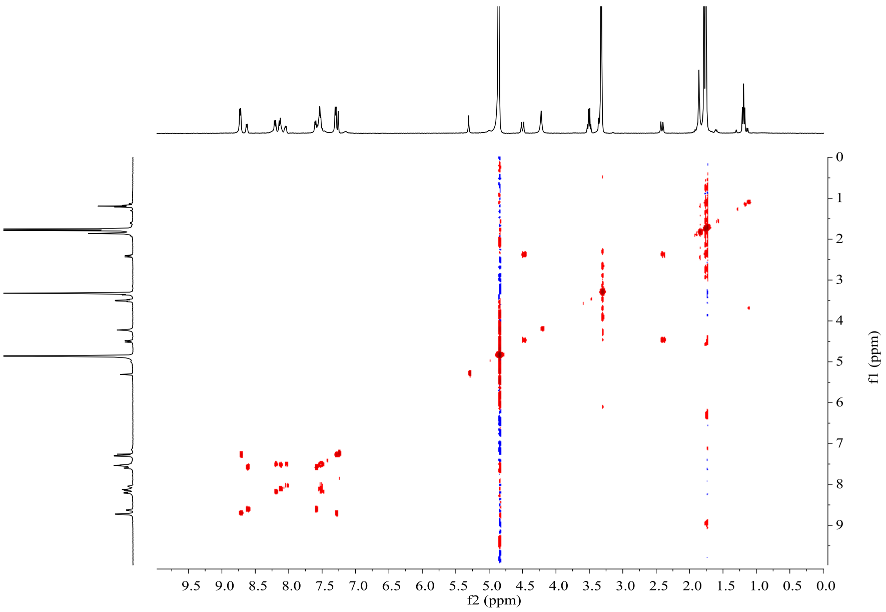

Supplementary Figure 30. ^{1}H COSY NMR (400 MHz, CD_3OD, ppm) for **2a+2b** (16.0 mM, with respect to Cp*Rh).

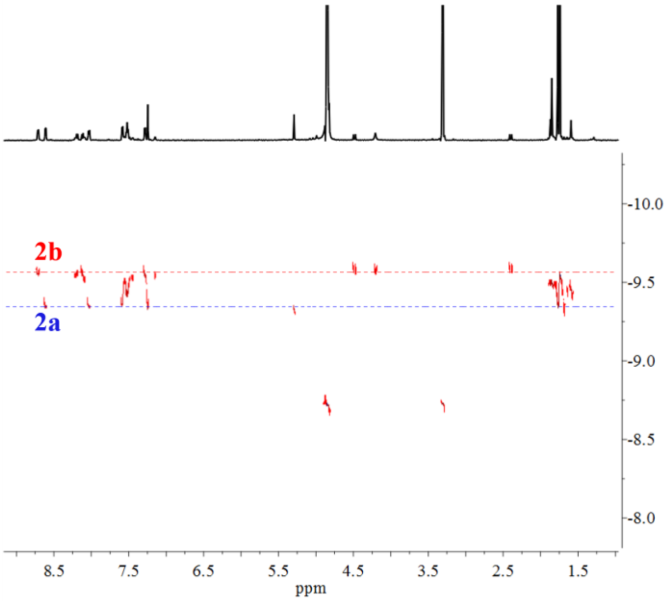

Supplementary Figure 31. ^{1}H DOSY NMR (500 MHz, CD_3OD, ppm) for **2a+2b** (16.0 mM, with respect to Cp*Rh). This DOSY spectra showed that tetranuclear macrocycle **2a** and trefoil knot **2b** existed at the same time in a medium-concentration solution Diffusion coefficient for **2a**: 4.7×10^{-10} m^2s^{-1} and **2b**: 2.8×10^{-10} m^2s^{-1}.

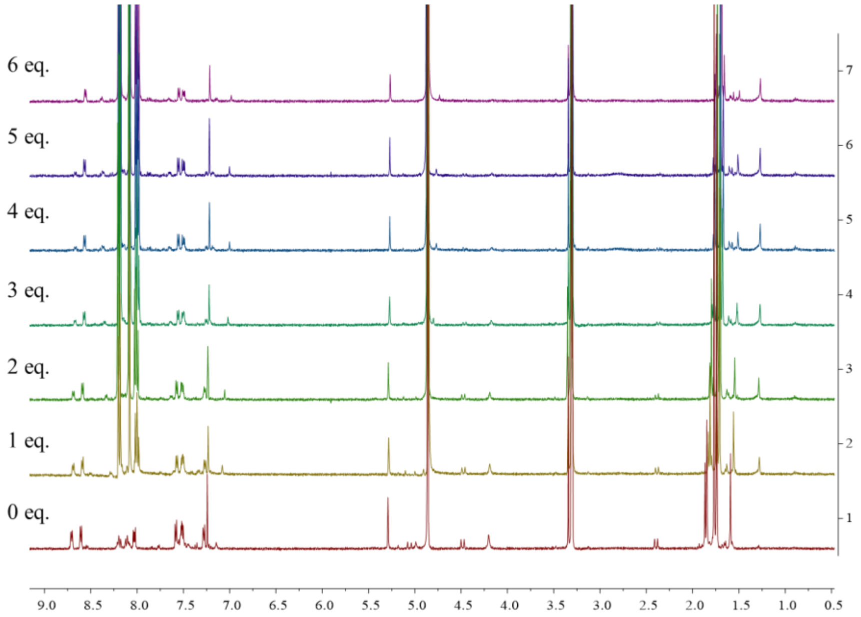

Supplementary Figure 32. ^{1}H NMR (400 MHz, CD_3OD, ppm) for **2a**+**2b** with increasing proportion of monomeric macrocycle **2a** upon addition of pyrene from 0 eq. to 6 eq. (12.0 mM, with respect to Cp*Rh).

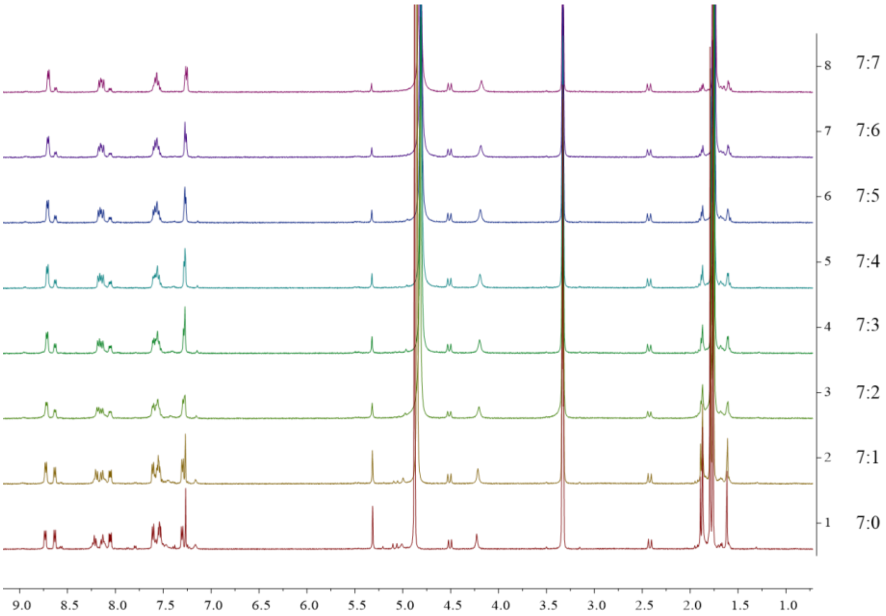

Supplementary Figure 33. ^{1}H NMR (400 MHz, CD_3OD, ppm) showing transformation of mixture of **2a**+**2b** into almost pure trefoil knot **2b** upon changing solvent ratio (CD_3OD:D_2O) from 7:0 to 7:7. (12.0 mM, with respect to Cp*Rh).

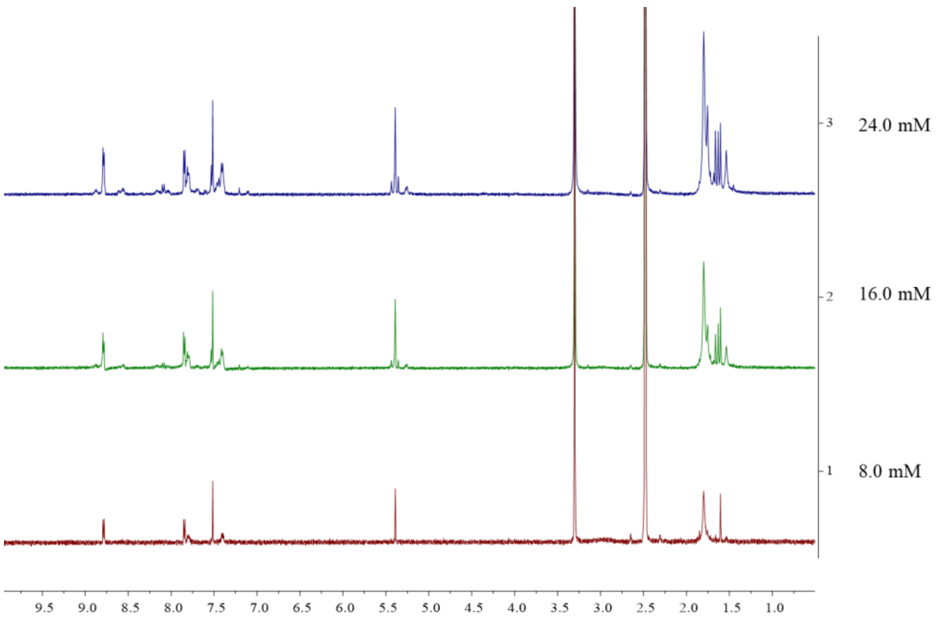

Supplementary Figure 34. ^{1}H NMR (400 MHz, DMSO, ppm) for **2a+2b** (8.0 mM to 24.0 mM, with respect to Cp*Rh).

4.5 NMR spectrum for 3

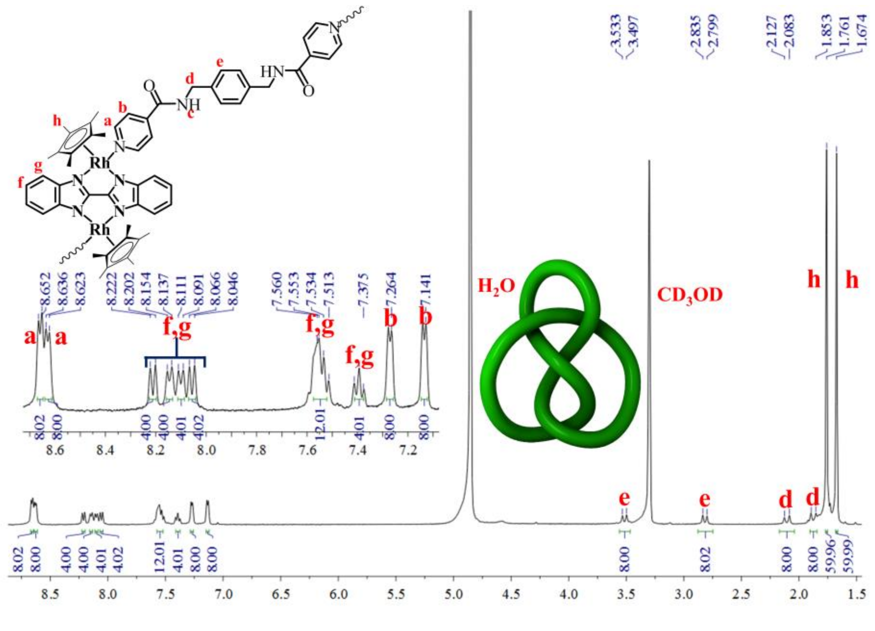

Supplementary Figure 35. ^{1}H NMR (400 MHz, CD_3OD, ppm) for **3** (Eight protons signals from NH (c) in the 1H NMR spectrum of **3** are shielded with respect to that of ligand **L2**)[1] (10.0 mM, with respect to Cp*Rh).

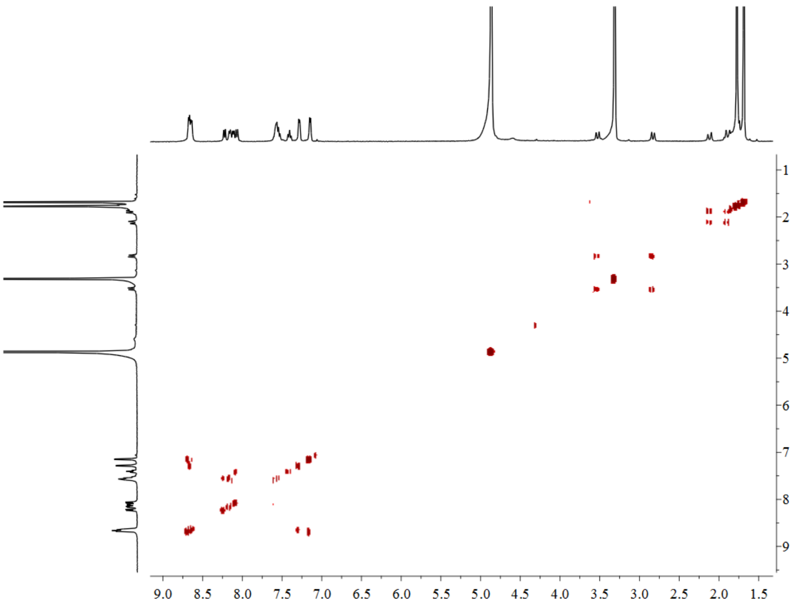

Supplementary Figure 36. ^{1}H COSY NMR (400 MHz, CD_3OD, ppm) for **3** (10.0 mM, with respect to Cp*Rh)**.** The ^{1}H NMR signals at 3.52 ppm and 2.82 ppm have a coupling interaction and they belong to phenyl protons. In addition, the ^{1}H NMR signals at 2.11 ppm and 1.87 ppm also have a coupling interaction, which belong to benzyl protons. Large upfield shifts of phenyl and benzyl protons indicate tight π-π stacking between phenyl and pyridyl groups, which contributes to the formation of Figure-Eight knot **3** and the stable structure of **3** results in the splitting of phenyl and benzyl protons.

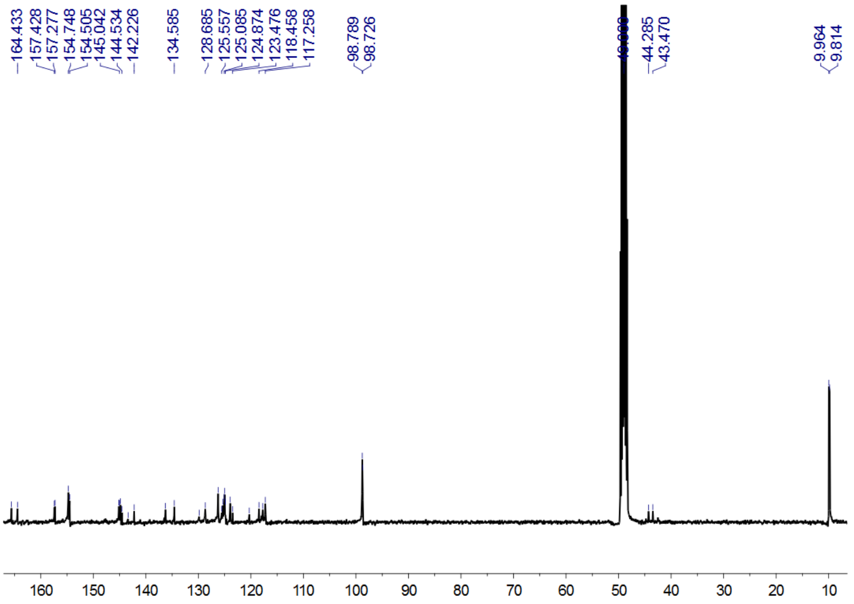

Supplementary Figure 37. $^{13}C\{^1H\}$ NMR (101 MHz, CD_3OD, ppm) for **3** (10.0 mM, with respect to Cp*Rh)**.**

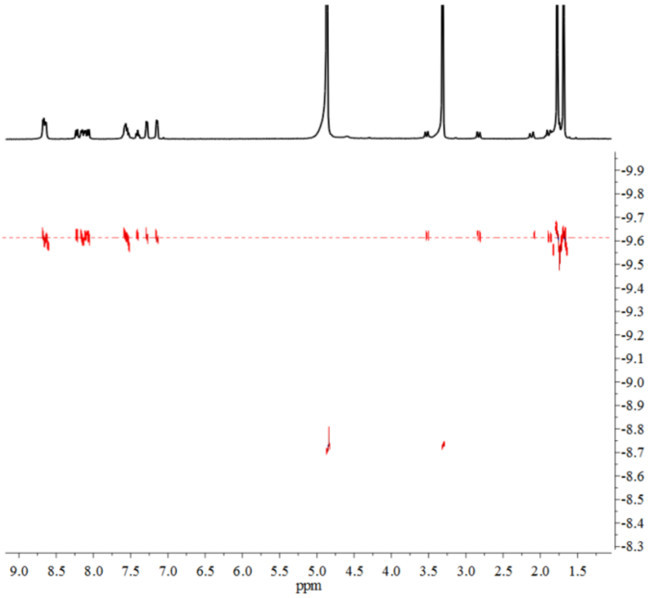

Supplementary Figure 38. ^{1}H DOSY NMR (500 MHz, CD_3OD, ppm) for **3.** (10.0 mM, with respect to Cp*Rh) Diffusion coefficient: 2.5×10^{-10} m^2s^{-1}.

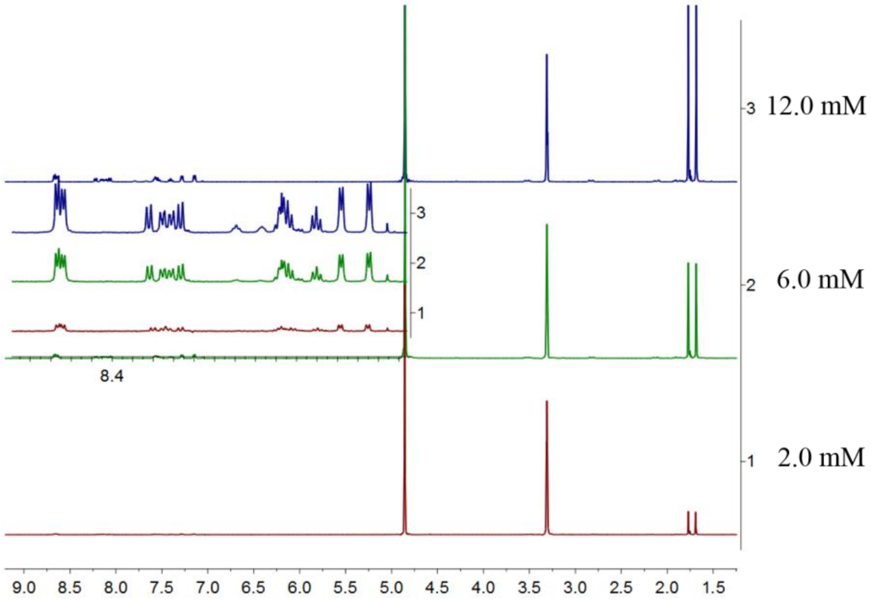

Supplementary Figure 39. ^{1}H NMR (400 MHz, CD_3OD, ppm) for **3** (2.0 mM to 12.0 mM, with respect to Cp*Rh)

4.6 NMR spectrum for 4

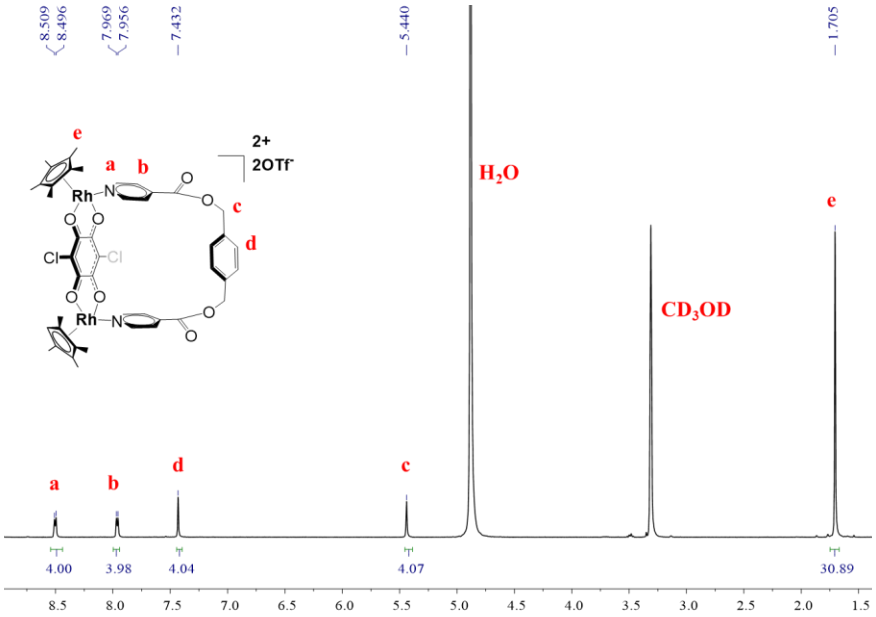

Supplementary Figure 40. ^{1}H NMR (400 MHz, CD3OD, ppm) for **4** (10.0 mM, with respect to Cp*Rh)**.**

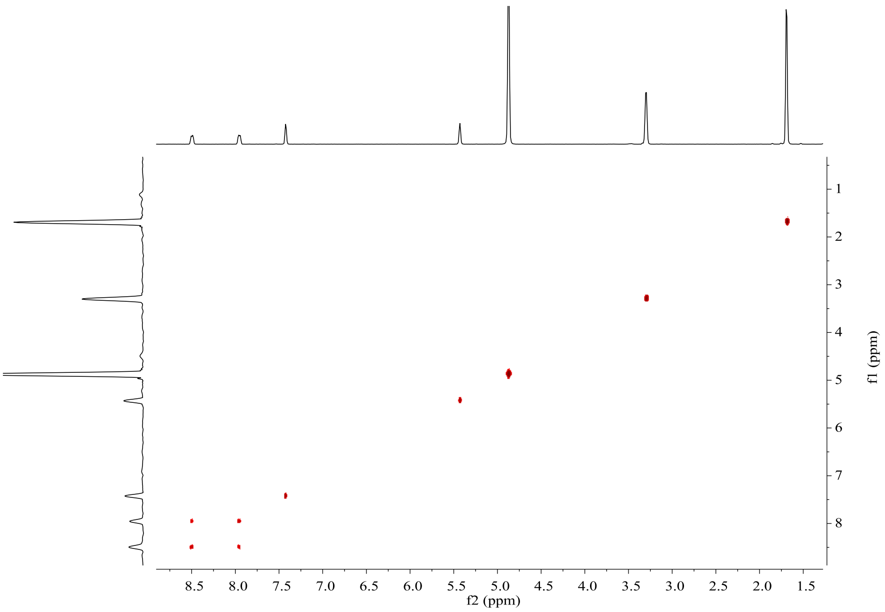

Supplementary Figure 41. ^{1}H COSY NMR (400 MHz, CD3OD, ppm) for **4** (20.0 mM, with respect to Cp*Rh)**.**

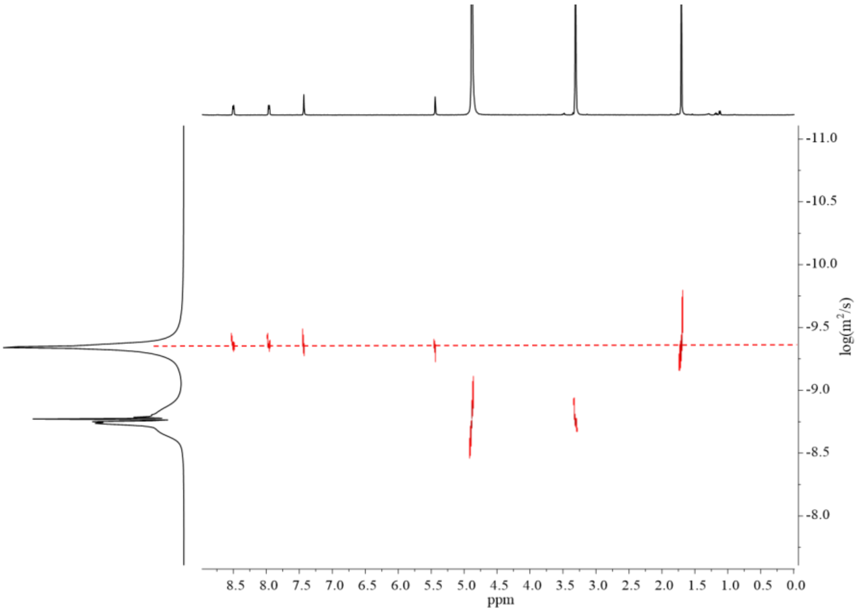

Supplementary Figure 42. ^{1}H DOSY NMR (400 MHz, CD_3OD, ppm) for **4** (10.0 mM, with respect to Cp*Rh).

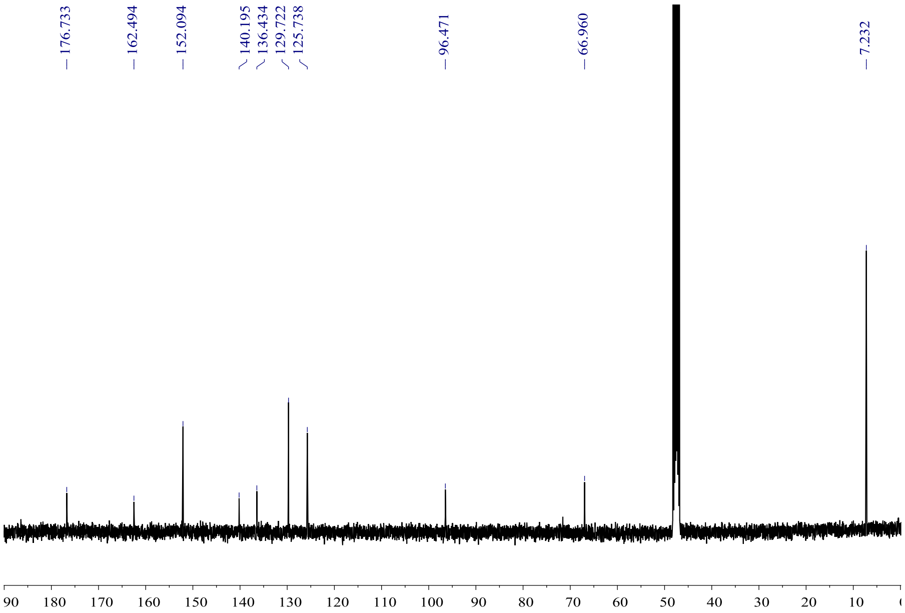

Supplementary Figure 43. $^{13}C\{^{1}H\}$ NMR (101 MHz, CD_3OD, ppm) for **4** (10.0 mM, with respect to Cp*Rh).

4.7 NMR spectrum for 5

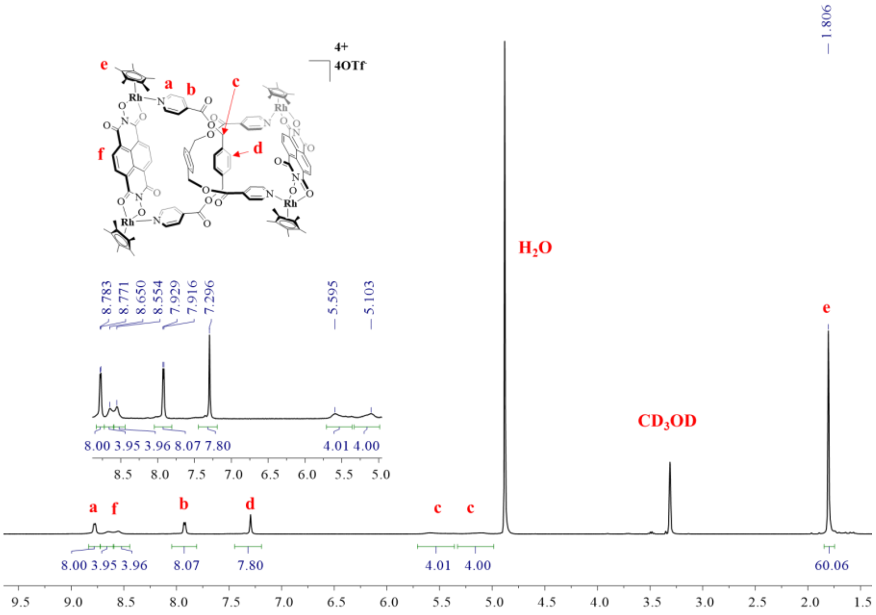

Supplementary Figure 44. ^{1}H NMR (400 MHz, CD_3OD, ppm) for **5** (20.0 mM, with respect to Cp*Rh).

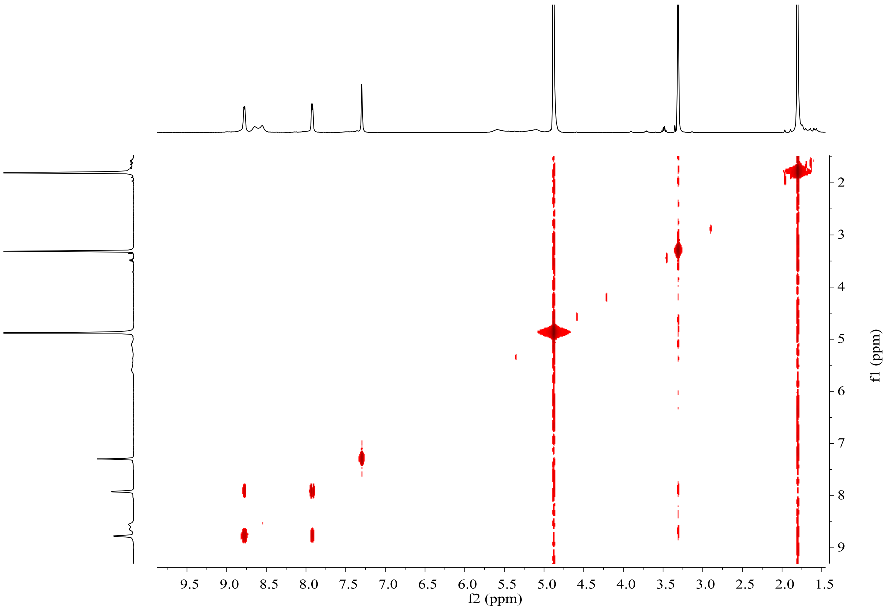

Supplementary Figure 45. ^{1}H COSY NMR (400 MHz, CD_3OD, ppm) for **5** (20.0 mM, with respect to Cp*Rh).

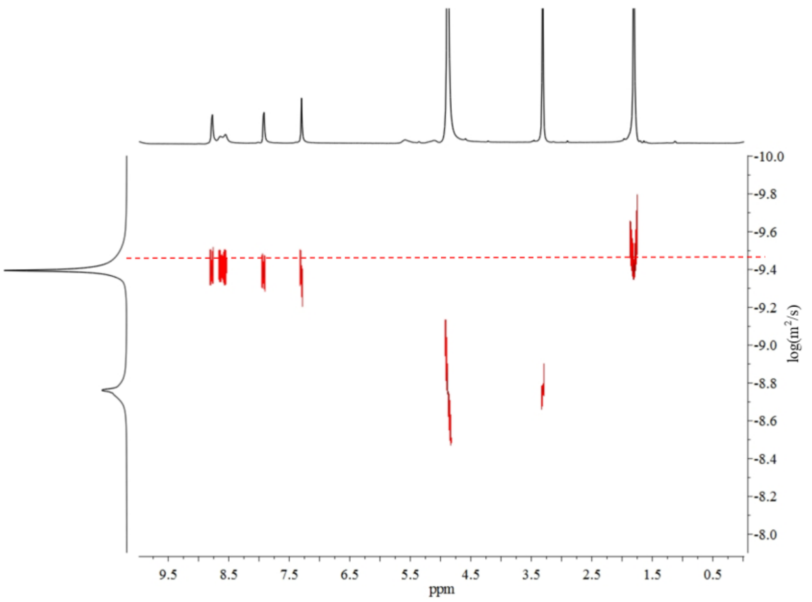

Supplementary Figure 46. ^{1}H DOSY NMR (400 MHz, CD_3OD, ppm) for **5** (20.0 mM, with respect to Cp*Rh)**.**

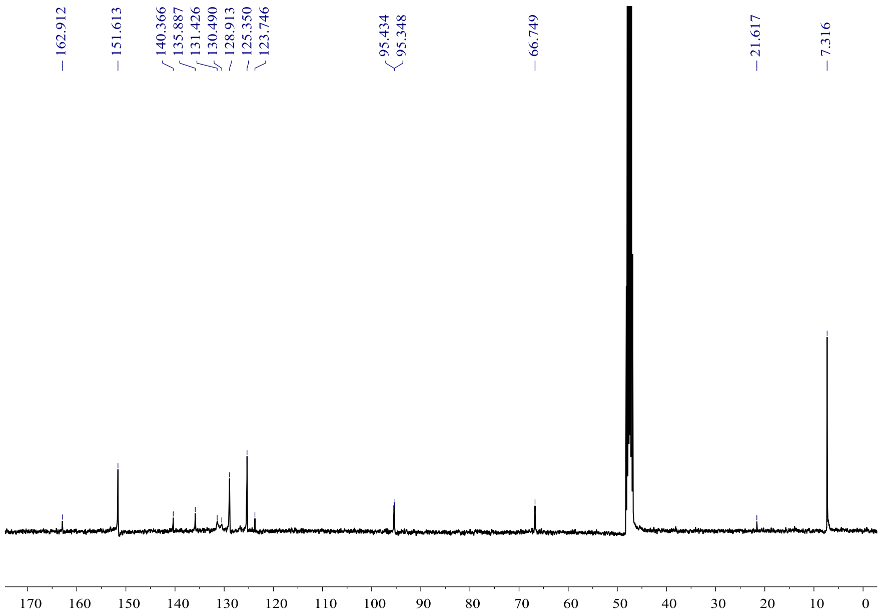

Supplementary Figure 47. $^{13}C\{^1H\}$ NMR (101 MHz, CD_3OD, ppm) for **5** (20.0 mM, with respect to Cp*Rh)**.**

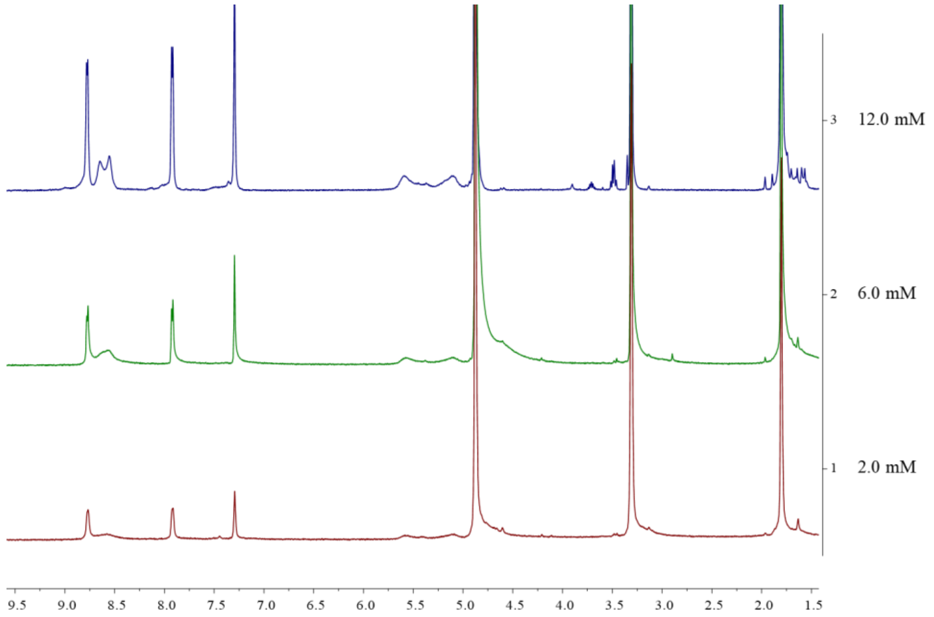

Supplementary Figure 48. ^{1}H NMR (400 MHz, CD_3OD, ppm) for **5** (2.0 mM to 12.0 mM, with respect to Cp*Rh).

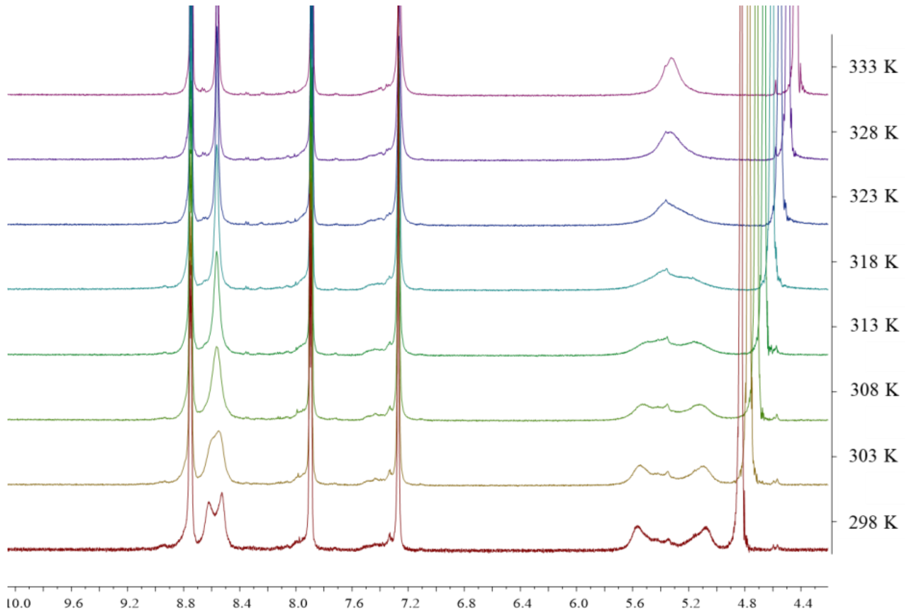

Supplementary Figure 49. Partial variable temperature ^{1}H NMR spectra (500 MHz, ppm) of complex **5** in CD_3OD solution between 298 K and 333 K (20.0 mM, with respect to Cp*Rh).

5. Supplementary Figures: ESI-MS spectra

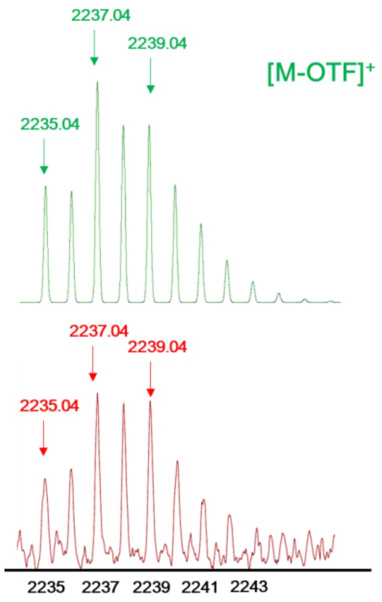

Supplementary Figure 50. Theoretical (top) and experimental (bottom) ESI-MS spectra of complex **1**.

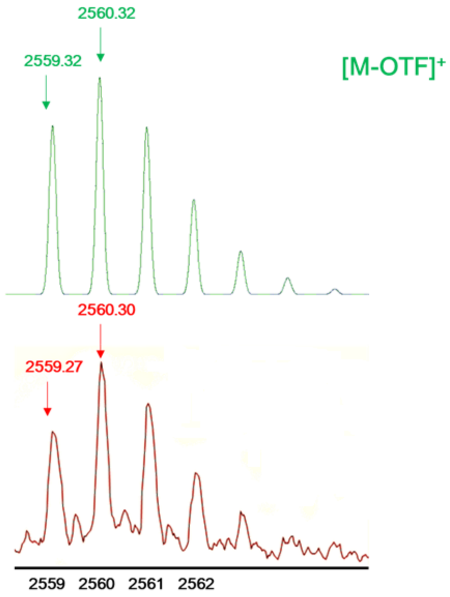

Supplementary Figure 51. Theoretical (top) and experimental (bottom) ESI-MS spectra of complex **2a**.

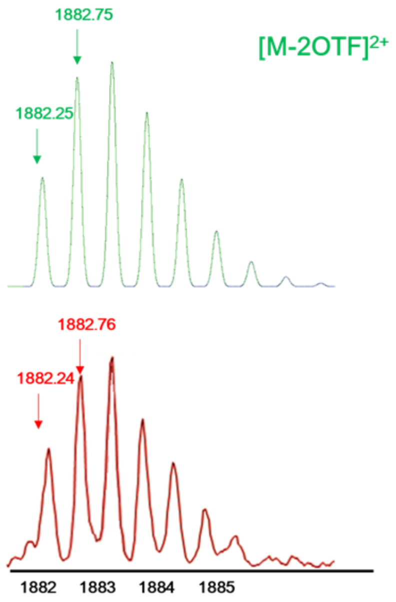

Supplementary Figure 52. Theoretical (top) and experimental (bottom) ESI-MS spectra of complex **2b**.

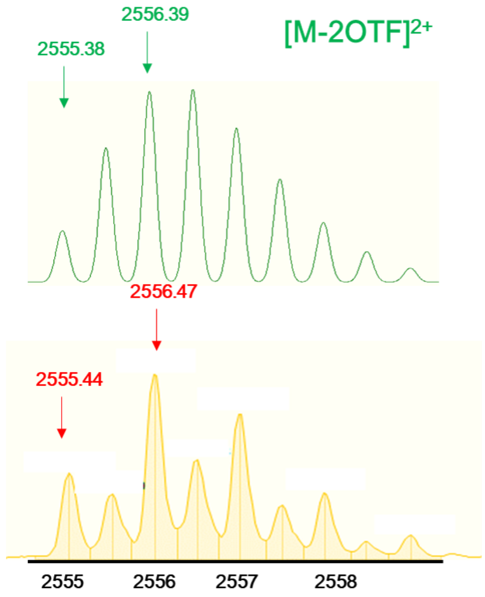

Supplementary Figure 53. Theoretical (top) and experimental (bottom) ESI-MS spectra of complex **3**.

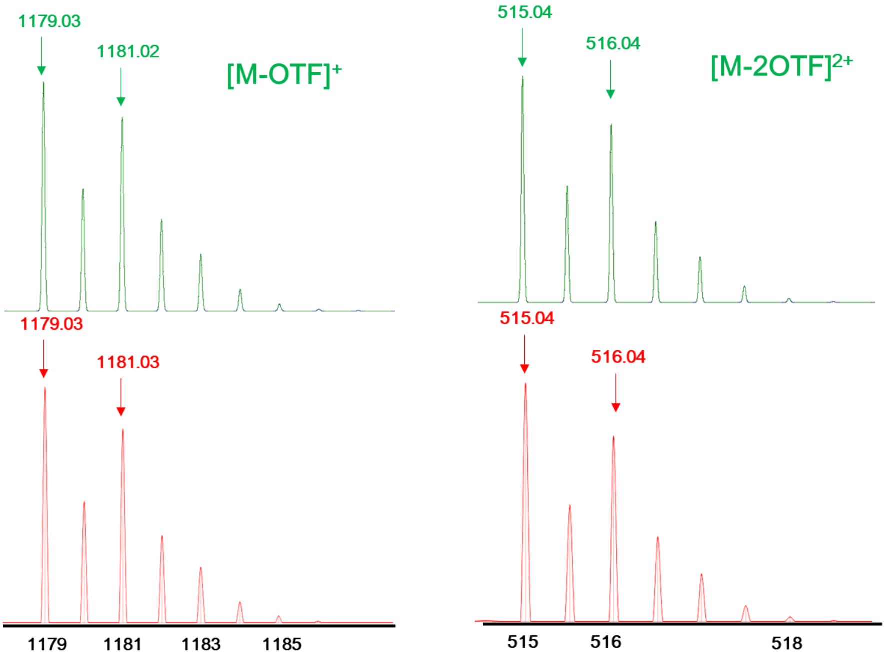

Supplementary Figure 54. Theoretical (top) and experimental (bottom) ESI-MS spectra of complex **4**.

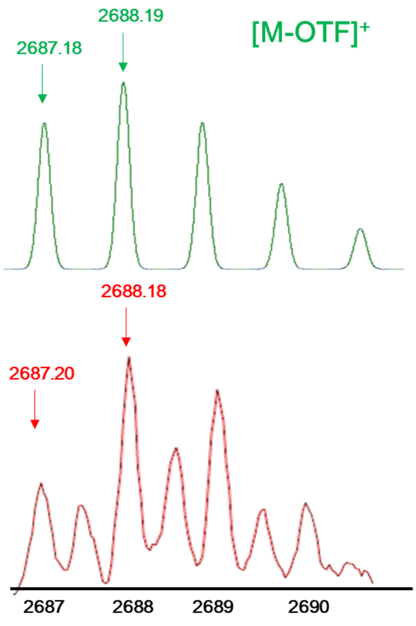

Supplementary Figure 55. Theoretical (top) and experimental (bottom) ESI-MS spectra of complex **5**.

6. Supplementary Notes: Computational details

All density functional theory (DFT) calculations were carried out using the SIESTA package with numerical atomic orbital basis sets and Troullier-Martins norm-conserving pseudopotentials.[2,3] The DFT functional utilized is the PBE functional,[4] a generalized gradient approximation DFT method. A double-ζ plus polarization (DZP) basis set was employed. The orbital-confining cutoff radii were determined from an energy shift of 0.01 eV. The energy cutoff for the real space grid used to represent the density was set as 150 Ry. The molecule was placed in the center of a cubic supercell with a length of 50 Å for each edge. Only Γ-point was used to sample the Brillouin zone in our calculations due to the large lattice parameter of the supercell. All atoms were relaxed until all the Cartesian forces on the atoms were lower than 0.02 eV/Bohr. To investigate the role of the π-π stacking interaction and other long-range interactions in the formation of these supermolecules, we optimized all the structures with or without the long-range dispersion correction of Grimme.[5] To account for entropy effect, all the structures were re-optimized and performed harmonic vibrational frequency analysis with the PM6 empirical method [6] to provide thermal correction energies at 298.15 K.

Supplementary Table 1. Energetic results at 298.15 K for the formation of **2a** (monomeric ring) and **2b** (trefoil knot) calculated with the PBE density functional method.

**Complex**	**ΔH (kcal/mol)**	**ΔG (kcal/mol)**
2_3mo (monomer like 4)		
2a	-10.4	-1.4
2b	-104.2	-77.4
After removing π-π interaction		
2a	16.5	25.6
After removing π-π interaction		
2b	-50.3	-23.5
Contribution of π-π interaction		
2a		-26.9
Contribution of π-π interaction		
2b		-53.9

Supplementary Table 2. Energetic results at 298.15 K for the formation of **5**([2]catenane) calculated with the PBE density functional method.

**Complex**	**ΔH (kcal/mol)**	**ΔG (kcal/mol)**
4_3mo (monomer like 4)		
5 ([2]catenane)	-48.7	-30.5
4_2a (monomeric ring like 2a)	3.6	11.4
After removing π-interaction 5	-7.3	10.9
After removing π-interaction 5_2a	2.1	9.9
Contribution of π-π interaction 5		-41.4
Contribution of π-π interaction 5_2a		1.5

Geometries:

All geometries presented here were optimized with long-range dispersion correction, i.e. using the PBE-D method.

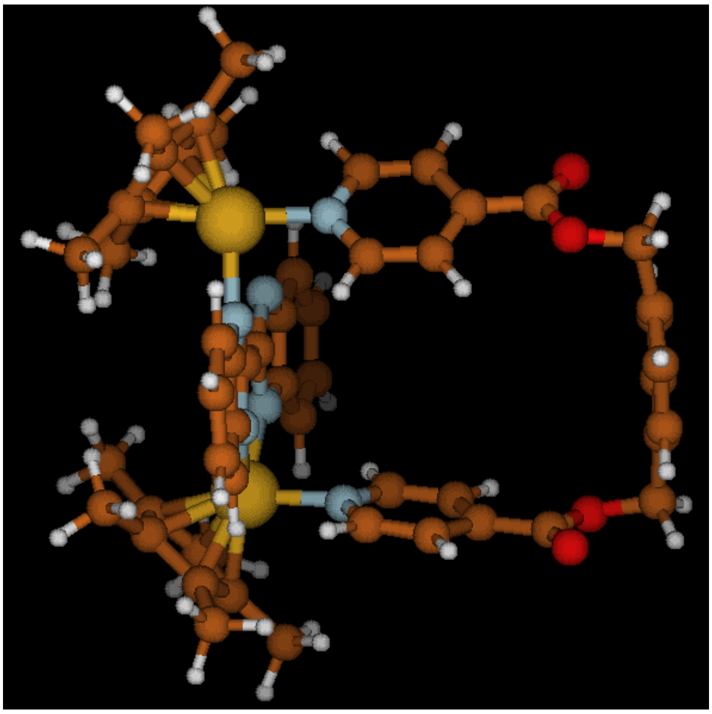

Supplementary Figure 56. Geometries of 2_3mo (monomer like **4**).

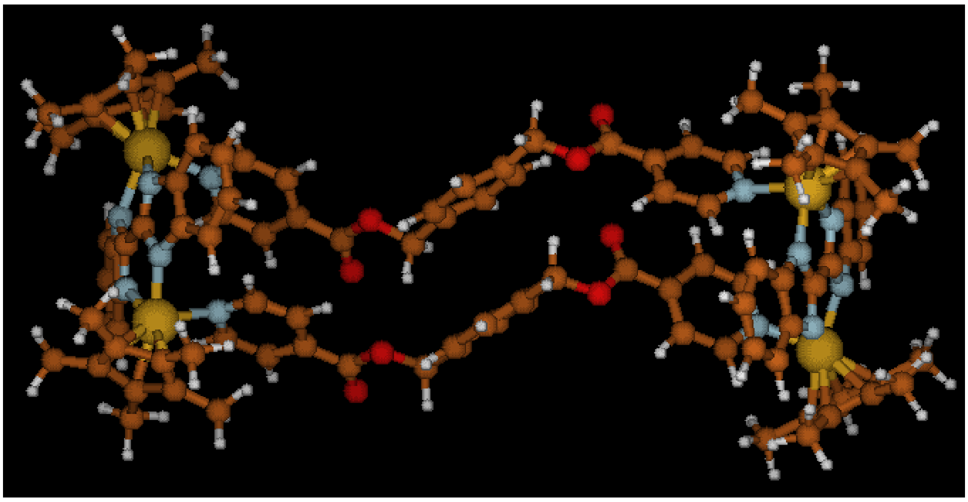

Supplementary Figure 57. Geometries of **2a**.

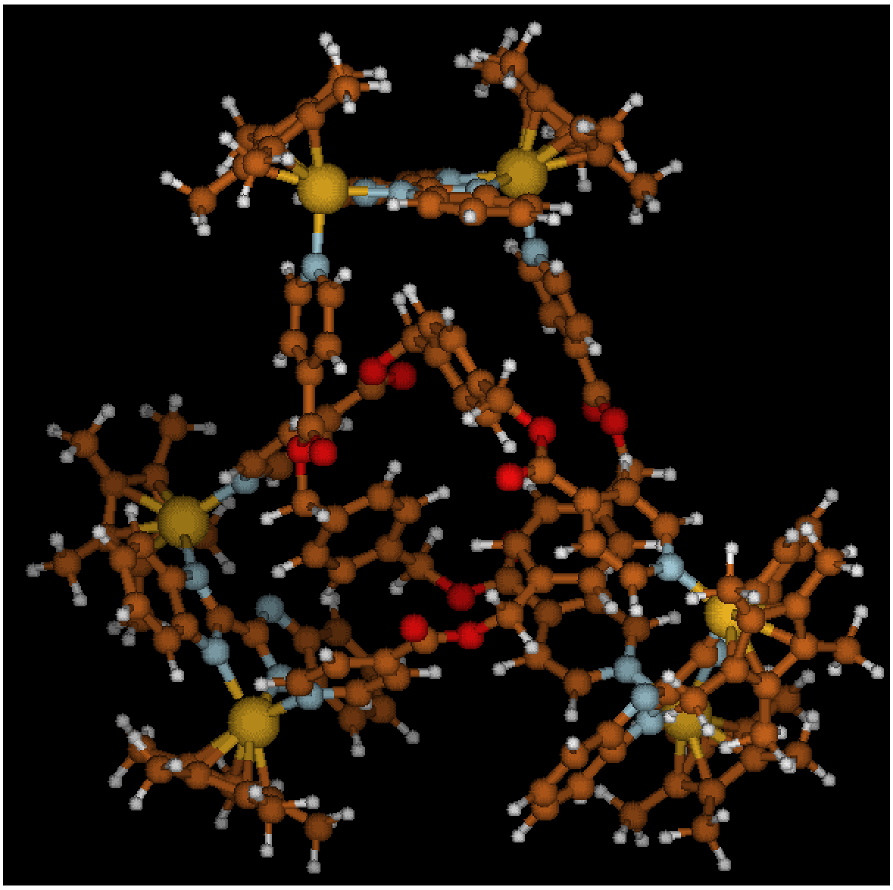

Supplementary Figure 58. Geometries of **2b**.

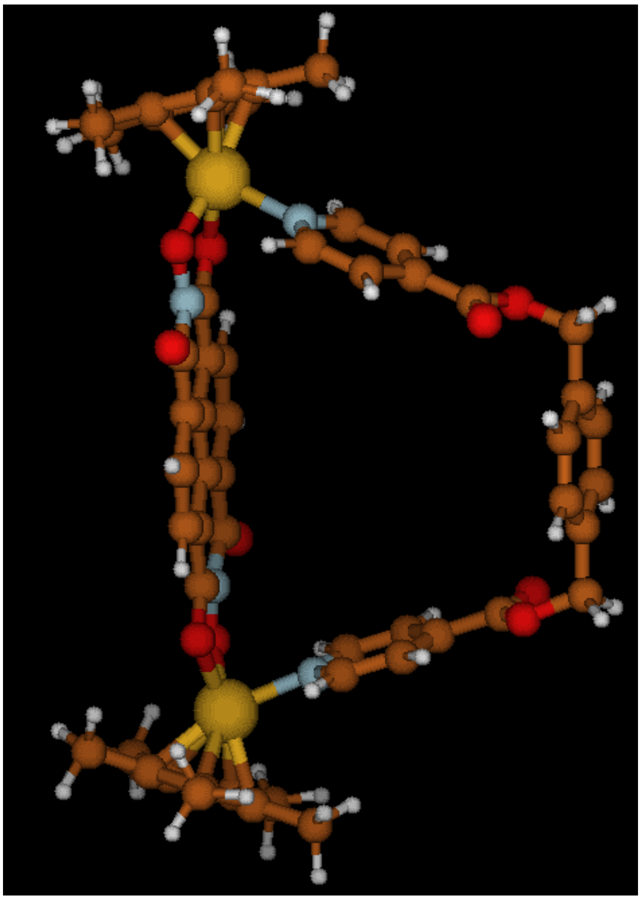

Supplementary Figure 59. Geometries of 4_3mo (monomer like **4**).

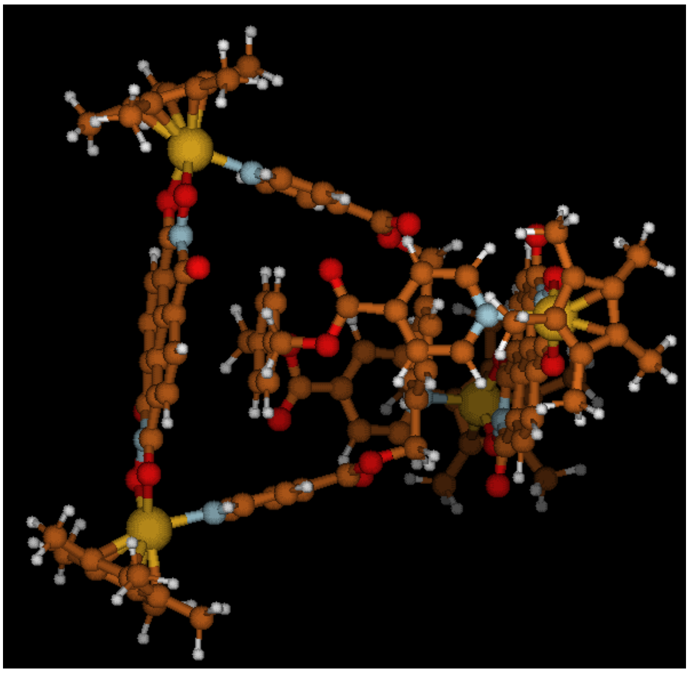

Supplementary Figure 60. Geometries of **5**.

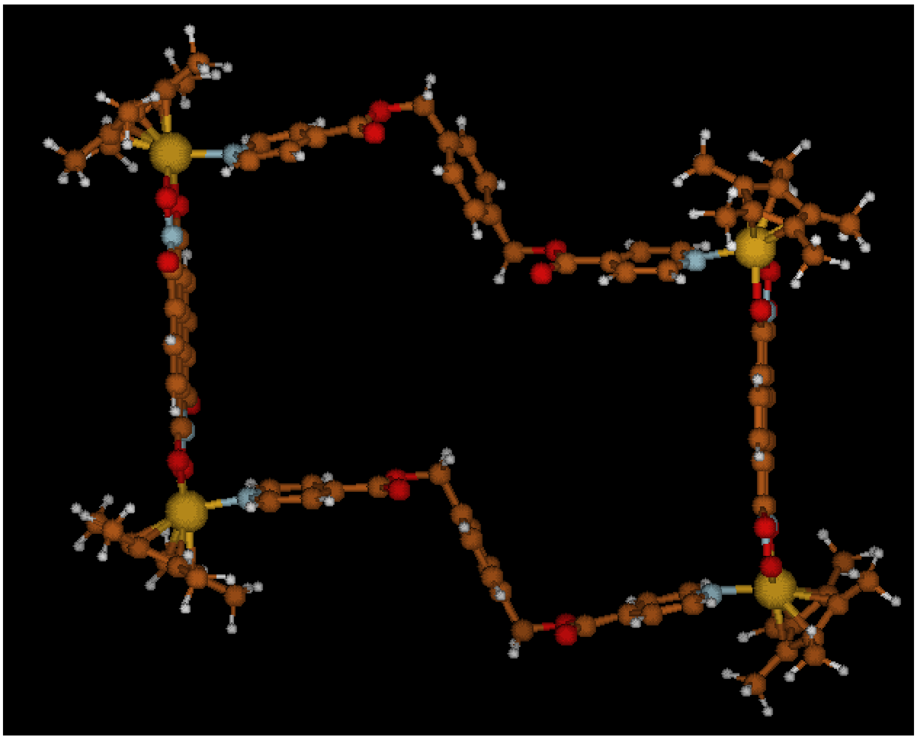

Supplementary Figure 61. Geometries of 5_2a (monomeric ring like **2a**).

7. Supplementary Tables: X-ray crystallography details

Single crystals of **1**, **2a**, **2b**, **3**, **3′**, **4** and **5**, suitable for X-ray diffraction study were obtained at room temperature. X-ray intensity data of **1** and **2b** were collected at 203 K, data of **2a** was collected at 193 K and data of **3**, **3′** and **4** were collected at 173 K on a CCD-Bruker SMART APEX system. In these data, the disordered solvent molecules which could not be restrained properly were removed using the SQUEEZE route.

In asymmetric unit of **1**, C44 and O12 were refined isotropically and other non-hydrogen atoms were refined anisotropically. 16 ISOR, 1 DELU and 1 DFIX instructions were used to restrain anions and solvents so that there were 98 restraints in the data. Hydrogen of one methanol molecule could not be found and others were put in calculated positions.

In asymmetric unit of **2a**, there were disordered anions and solvents (three triflate anion and four methanol molecules) which could not be restrained properly. Therefore, SQUEEZE algorithm was used to omit them. One pentamethylcyclopentadienyl ligand (Cp* for short) was disordered and it was divided into two parts (50:50). O30 and O31 were refined isotropically and other non-hydrogen atoms were refined anisotropically. 42 ISOR and 13 DFIX instructions were used to restrain anions, solvents and Cp* fragments so that there were 265 restraints in the data. Hydrogen of methanol molecules could not be found and others were put in calculated positions.

In asymmetric unit of **2b**, there were disordered anion and solvents (one triflate anion, four methanol and five water molecules) which could not be restrained properly. Therefore, SQUEEZE algorithm was used to omit them. One metalla-edge (Rh3 and corresponding Cp* fragment) was disordered and it was divided into two parts (44:56). F4, O11 and F5 were refined isotropically and other non-hydrogen atoms were refined anisotropically. 51 ISOR, 3 SIMU, 10 DELU and 26 DFIX instructions were used to restrain anions, ligands and Cp* fragments so that there were 488 restraints in the data.

In asymmetric unit of **3**, there were disordered solvent molecules (six methanol and five water molecules) which could not be restrained properly. Therefore, SQUEEZE algorithm was used to omit them. 6 ISOR and 2 DFIX instructions were used to restrain anions and solvents so that there were 38 restraints in the data. Hydrogen of methanol molecules could not be found and others were put in calculated positions.

In asymmetric unit of **3′**, there were disordered anions and solvents (two triflate anions, half of a di-isopropyl ether and half of a methanol molecules) which could not be restrained properly. Therefore,

SQUEEZE algorithm was used to omit them. 21 ISOR, 3 DELU and 4 DFIX instructions were used to restrain ligand and Cp* fragments so that there were 136 restraints in the data.

In asymmetric unit of **4**, there were disordered solvents (two and a half methanol molecules) which could not be restrained properly. Therefore, SQUEEZE algorithm was used to omit them. One triflate anion and one diethyl ether molecule were disordered and they were divided into two parts (65:35 for anion and 56:44 for Et2O). 20 ISOR and 9 DFIX instructions were used to restrain anions, solvent molecule and Cp* fragments so that there were 129 restraints in the data.

In asymmetric unit of **5**, there were one disordered triflate anion which could not be restrained properly. Therefore, SQUEEZE algorithm was used to omit them. Hydrogen of methanol and water molecules could not be found and others were put in calculated positions.

Supplementary Table 3. Crystal data and structure refinement for **1**.

Identification code	mo_71215a	
Empirical formula	C88 H108 Cl4 F12 N4 O24 Rh4 S4	
Formula weight	2515.46	
Temperature	203(2) K	
Wavelength	0.71073 Å	
Crystal system	Triclinic	
Space group	P-1	
Unit cell dimensions	a = 11.822(3) Å	= 114.063(4) °.
	b = 14.553(4) Å	= 99.531(4) °.
	c = 17.169(5) Å	= 92.220(4) °.
Volume	2641.5(12) $Å^3$	
Z	1	
Density (calculated)	1.581 Mg/m^3	
Absorption coefficient	0.884 mm^{-1}	
F(000)	1276	
Crystal size	0.280 x 0.250 x 0.190 mm^3	
Theta range for data collection	1.325 to 26.499 °.	
Index ranges	-14<=h<=13, -18<=k<=12, -21<=l<=21	
Reflections collected	17366	
Independent reflections	10682 [R(int) = 0.0450]	
Completeness to theta = 25.242 °	97.5 %	
Absorption correction	Semi-empirical from equivalents	
Max. and min. transmission	0.746 and 0.546	
Refinement method	Full-matrix least-squares on F^2	
Data / restraints / parameters	10682 / 98 / 633	
Goodness-of-fit on F^2	1.195	
Final R indices [I>2sigma(I)]	R1 = 0.1063, wR2 = 0.3321	
R indices (all data)	R1 = 0.1610, wR2 = 0.3863	
Extinction coefficient	n/a	
Largest diff. peak and hole	3.932 and -1.867 e.$Å^{-3}$	

$^aR_1 = \Sigma||F_o| - |F_c||$ (based on reflections with $Fo^2 > 2\sigma F^2$); $wR_2 = \{\Sigma[\omega(Fo^2 - Fc^2)^2]/\Sigma[\omega(Fo^2)^2]\}^{1/2}$; $w = 1/[\sigma^2 F_0^2 + (0.095P)^2]$; $P = [\max(Fo^2, 0) + 2Fc^2]/3$ (also with $Fo^2 > 2\sigma F^2$).

Supplementary Table 4. Crystal data and structure refinement for **2a**.

Identification code	171202fd1_a_sq	
Empirical formula	C120 H137 F12 N12 O26.50 Rh4 S4	
Formula weight	2939.29	
Temperature	193.0 K	
Wavelength	1.54178 Å	
Crystal system	Triclinic	
Space group	P-1	
Unit cell dimensions	a = 17.1132(10) Å	= 84.453(4)°.
	b = 28.8549(18) Å	= 75.591(3)°.
	c = 29.0193(19) Å	= 72.958(3)°.
Volume	13265.2(15) $Å^3$	
Z	4	
Density (calculated)	1.472 Mg/m^3	
Absorption coefficient	5.309 mm^{-1}	
F(000)	6020	
Crystal size	0.57 x 0.32 x 0.05 mm^3	
Theta range for data collection	2.216 to 66.998°.	
Index ranges	-17<=h<=20, -34<=k<=33, -34<=l<=34	
Reflections collected	103205	
Independent reflections	44498 [R(int) = 0.0857]	
Completeness to theta = 66.998°	94.0 %	
Absorption correction	Semi-empirical from equivalents	
Max. and min. transmission	0.4684 and 0.1998	
Refinement method	Full-matrix least-squares on F^2	
Data / restraints / parameters	44498 / 265 / 2567	
Goodness-of-fit on F^2	1.058	
Final R indices [I>2sigma(I)]	R1 = 0.1286, wR2 = 0.3099	
R indices (all data)	R1 = 0.1657, wR2 = 0.3344	
Extinction coefficient	n/a	
Largest diff. peak and hole	2.771 and -1.650 e.$Å^{-3}$	

$^aR_1 = \Sigma||F_o| - |F_c||$ (based on reflections with $Fo^2 > 2\sigma F^2$); $wR_2 = \{\Sigma[\omega(Fo^2 - Fc^2)^2]/\Sigma[\omega(Fo^2)^2]\}^{1/2}$; $w = 1/[\sigma^2 F_0^2 + (0.095P)^2]$; $P = [\max(Fo^2, 0) + 2Fc^2]/3$ (also with $Fo^2 > 2\sigma F^2$).

Supplementary Table 5. Crystal data and structure refinement for **2b**.

Identification code	mo_71123b_0m_sq	
Empirical formula	C176 H214 F18 N18 O48 Rh6 S6	
Formula weight	4501.46	
Temperature	203(2) K	
Wavelength	0.71073 Å	
Crystal system	Monoclinic	
Space group	C2/c	
Unit cell dimensions	a = 36.727(9) Å	= 90 °.
	b = 25.247(7) Å	= 91.905(5) °.
	c = 21.516(6) Å	= 90 °.
Volume	19940(9) Å^3	
Z	4	
Density (calculated)	1.499 Mg/m^3	
Absorption coefficient	0.645 mm^{-1}	
F(000)	9232	
Crystal size	0.250 x 0.220 x 0.180 mm^3	
Theta range for data collection	0.979 to 25.249 °.	
Index ranges	-34<=h<=44, -30<=k<=30, -25<=l<=25	
Reflections collected	60020	
Independent reflections	18005 [R(int) = 0.1171]	
Completeness to theta = 25.242 °	99.7 %	
Absorption correction	Semi-empirical from equivalents	
Max. and min. transmission	0.746 and 0.557	
Refinement method	Full-matrix least-squares on F^2	
Data / restraints / parameters	18005 / 474 / 1102	
Goodness-of-fit on F^2	1.180	
Final R indices [I>2sigma(I)]	R1 = 0.1263, wR2 = 0.3448	
R indices (all data)	R1 = 0.2175, wR2 = 0.4166	
Extinction coefficient	n/a	
Largest diff. peak and hole	2.101 and -0.679 e.Å^{-3}	

$^aR_1 = \Sigma||F_o| - |F_c||$ (based on reflections with $Fo^2 > 2\sigma F^2$); $wR_2 = \{\Sigma[\omega(Fo^2 - Fc^2)^2]/\Sigma[\omega(Fo^2)^2]\}^{1/2}$; $w = 1/[\sigma^2 F_0^2 + (0.095P)^2]$; $P = [\max(Fo^2, 0) + 2Fc^2]/3$ (also with $Fo^2 > 2\sigma F^2$).

Supplementary Table 6. Crystal data and structure refinement for **3**.

Identification code	ga_80503c_a_sq	
Empirical formula	C256 H392 F24 N32 O84 Rh8 S8	
Formula weight	6797.75	
Temperature	173(2) K	
Wavelength	1.34138 Å	
Crystal system	Tetragonal	
Space group	$I4_1/a$	
Unit cell dimensions	a = 42.8137(10) Å	= 90°.
	b = 42.8137(10) Å	= 90°.
	c = 16.3559(3) Å	= 90°.
Volume	29980.6(15) $Å^3$	
Z	4	
Density (calculated)	1.506 Mg/m^3	
Absorption coefficient	3.258 mm^{-1}	
F(000)	14112	
Crystal size	0.430 x 0.220 x 0.220 mm^3	
Theta range for data collection	3.091 to 54.970°.	
Index ranges	-52<=h<=47, -51<=k<=52, -19<=l<=19	
Reflections collected	84018	
Independent reflections	14252 [R(int) = 0.0310]	
Completeness to theta = 53.594°	99.9 %	
Absorption correction	Semi-empirical from equivalents	
Max. and min. transmission	0.254 and 0.128	
Refinement method	Full-matrix least-squares on F^2	
Data / restraints / parameters	14250 / 38 / 785	
Goodness-of-fit on F^2	1.067	
Final R indices [I>2sigma(I)]	R1 = 0.0422, wR2 = 0.1207	
R indices (all data)	R1 = 0.0441, wR2 = 0.1223	
Extinction coefficient	n/a	
Largest diff. peak and hole	1.345 and -0.699 e.$Å^{-3}$	

$^aR_1 = \Sigma||F_o| - |F_c||$ (based on reflections with $Fo^2 > 2\sigma F^2$); $wR_2 = \{\Sigma[\omega(Fo^2 - Fc^2)^2]/\Sigma[\omega(Fo^2)^2]\}^{1/2}$; $w = 1/[\sigma^2 F_0^2 + (0.095P)^2]$; $P = [\max(Fo^2, 0) + 2Fc^2]/3$ (also with $Fo^2 > 2\sigma F^2$).

Supplementary Table 7. Crystal data and structure refinement for **3′**.

Identification code	ga_80709b_a_sq	
Empirical formula	C238 H260 F24 Ir8 N32 O36 S8	
Formula weight	6394.84	
Temperature	173(2) K	
Wavelength	1.34138 Å	
Crystal system	Tetragonal	
Space group	I-4	
Unit cell dimensions	a = 19.3043(11) Å	a= 90 °.
	b = 19.3043(11) Å	b= 90 °.
	c = 33.3141(18) Å	g = 90 °.
Volume	12414.7(16) Å3	
Z	2	
Density (calculated)	1.711 Mg/m3	
Absorption coefficient	6.280 mm-1	
F(000)	6320	
Crystal size	0.270 x 0.260 x 0.210 mm3	
Theta range for data collection	2.816 to 57.140 °.	
Index ranges	-23<=h<=24, -24<=k<=24, -41<=l<=41	
Reflections collected	52291	
Independent reflections	12493 [R(int) = 0.0539]	
Completeness to theta = 53.594 °	99.8 %	
Absorption correction	Semi-empirical from equivalents	
Max. and min. transmission	0.751 and 0.328	
Refinement method	Full-matrix least-squares on F2	
Data / restraints / parameters	12493 / 136 / 580	
Goodness-of-fit on F2	1.050	
Final R indices [I>2sigma(I)]	R1 = 0.0533, wR2 = 0.1379	
R indices (all data)	R1 = 0.0536, wR2 = 0.1382	
Absolute structure parameter	0.21(2)	
Extinction coefficient	n/a	
Largest diff. peak and hole	2.322 and -1.334 e.Å-3	

$^{a}R_1 = \Sigma||F_o| - |F_c||$ (based on reflections with $Fo^2 > 2\sigma F^2$); $wR_2 = \{\Sigma[\omega(Fo^2 - Fc^2)^2]/\Sigma[\omega(Fo^2)^2]\}^{1/2}$; $w = 1/[\sigma^2 F_0^2 + (0.095P)^2]$; $P = [\max(Fo^2, 0) + 2Fc^2]/3$ (also with $Fo^2 > 2\sigma F^2$).

Supplementary Table 8. Crystal data and structure refinement for **4**.

Identification code	mo_70324b	
Empirical formula	C54.50 H66 Cl2 F6 N2 O17.50 Rh2 S2	
Formula weight	1483.93	
Temperature	173(2) K	
Wavelength	0.71073 Å	
Crystal system	Monoclinic	
Space group	$P2_1/c$	
Unit cell dimensions	a = 14.8514(18) Å	= 90 °.
	b = 30.272(4) Å	= 111.817(2) °.
	c = 15.4957(19) Å	= 90 °.
Volume	6467.6(14) $Å^3$	
Z	4	
Density (calculated)	1.524 Mg/m^3	
Absorption coefficient	0.741 mm^{-1}	
F(000)	3028	
Crystal size	0.830 x 0.380 x 0.100 mm^3	
Theta range for data collection	1.477 to 27.850 °.	
Index ranges	-19<=h<=12, -39<=k<=39, -20<=l<=19	
Reflections collected	44119	
Independent reflections	15292 [R(int) = 0.0378]	
Completeness to theta = 25.242 °	99.6 %	
Absorption correction	Semi-empirical from equivalents	
Max. and min. transmission	0.746 and 0.618	
Refinement method	Full-matrix least-squares on F^2	
Data / restraints / parameters	15292 / 129 / 860	
Goodness-of-fit on F^2	1.092	
Final R indices [I>2sigma(I)]	R1 = 0.0720, wR2 = 0.1776	
R indices (all data)	R1 = 0.0874, wR2 = 0.1867	
Extinction coefficient	n/a	
Largest diff. peak and hole	3.697 and -2.054 e.$Å^{-3}$	

$aR_1 = \Sigma||F_o| - |F_c||$ (based on reflections with $Fo^2 > 2\sigma F^2$); $wR_2 = \{\Sigma[\omega(Fo^2 - Fc^2)^2]/\Sigma[\omega(Fo^2)^2]\}^{1/2}$; $w = 1/[\sigma^2 F_0^2 + (0.095P)^2]$; $P = [\max(Fo^2, 0) + 2Fc^2]/3$ (also with $Fo^2 > 2\sigma F^2$).

Supplementary Table 9. Crystal data and structure refinement for **5**.

Identification code	mo_70411ba_sq	
Empirical formula	C58 H62 F6 N4 O19 Rh2 S2	
Formula weight	1503.05	
Temperature	173(2) K	
Wavelength	0.71073 Å	
Crystal system	Orthorhombic	
Space group	Pban	
Unit cell dimensions	a = 24.072(3) Å	= 90 °.
	b = 15.2230(17) Å	= 90 °.
	c = 17.711(2) Å	= 90 °.
Volume	6490.2(12) $Å^3$	
Z	4	
Density (calculated)	1.538 Mg/m^3	
Absorption coefficient	0.663 mm^{-1}	
F(000)	3064	
Crystal size	0.300 x 0.210 x 0.080 mm^3	
Theta range for data collection	1.956 to 27.558 °.	
Index ranges	-28<=h<=31, -19<=k<=19, -22<=l<=21	
Reflections collected	45595	
Independent reflections	7460 [R(int) = 0.0889]	
Completeness to theta = 25.242 °	99.7 %	
Absorption correction	Semi-empirical from equivalents	
Max. and min. transmission	0.746 and 0.664	
Refinement method	Full-matrix least-squares on F^2	
Data / restraints / parameters	7460 / 0 / 344	
Goodness-of-fit on F^2	1.006	
Final R indices [I>2sigma(I)]	R1 = 0.0684, wR2 = 0.1827	
R indices (all data)	R1 = 0.1167, wR2 = 0.2090	
Extinction coefficient	n/a	
Largest diff. peak and hole	1.719 and -0.857 e.$Å^{-3}$	

$^aR_1 = \Sigma||F_o| - |F_c||$ (based on reflections with $Fo^2 > 2\sigma F^2$); $wR_2 = \{\Sigma[\omega(Fo^2 - Fc^2)^2]/\Sigma[\omega(Fo^2)^2]\}^{1/2}$; $w = 1/[\sigma^2 F_0^2 + (0.095P)^2]$; $P = [\max(Fo^2, 0) + 2Fc^2]/3$ (also with $Fo^2 > 2\sigma F^2$).

8. Supplementary references

1. Danon, J. J., Krüger, A., Leigh, D. A., Lemonnier, J.-F., Stephens, A. J., Vitorica-Yrezabal, I. J., Woltering, S. L. Braiding a molecular knot with eight crossings. *Science*., **355**, 159-162 (2017).
2. Junquera, J., Paz, O., Sanchez-Portal, D., Artacho, E. Numerical atomic orbitals for linear-scaling calculations. *Phys. Rev. B*. **64**, 235111 (2001).
3. Troullier, N., Martins, J. L. Efficient pseudopotentials for plane-wave calculations. *Phys. Rev. B*. **43**, 1993 (1991).
4. Perdew, J. P., Burke, K., Ernzerhof, M. Phys. Generalized Gradient Approximation Made Simple. *Rev. Lett.* **77**, 3865 (1996).
5. Grimme, S. J. Semiempirical GGA-type density functional constructed with a long-range dispersion correction. *Comput. Chem*. **27**, 1787-1799 (2006).
6. Stewart, J. J. P. Optimization of parameters for semiempirical methods V: Modification of NDDO approximations and application to 70 elements. *J. Mol. Model*. **13**, 1173-1213 (2007).
